# Mobile Health Physical Activity Intervention Preferences in Cancer Survivors: A Qualitative Study

**DOI:** 10.2196/mhealth.6970

**Published:** 2017-01-24

**Authors:** Michael C Robertson, Edward Tsai, Elizabeth J Lyons, Sanjana Srinivasan, Maria C Swartz, Miranda L Baum, Karen M Basen-Engquist

**Affiliations:** ^1^ Center for Energy Balance Department of Behavioral Science MD Anderson Cancer Center Houston, TX United States; ^2^ Health Promotion & Behavioral Sciences University of Texas School of Public Health Houston, TX United States; ^3^ Department of Nutrition and Metabolism University of Texas Medical Branch Galveston, TX United States; ^4^ Epidemiology, Human Genetics & Environmental Sciences University of Texas School of Public Health Houston, TX United States; ^5^ Center for Recovery, Physical Activity and Nutrition Division of Rehabilitation Sciences University of Texas Medical Branch Galveston, TX United States

**Keywords:** mHealth, physical activity, survivors, technology, focus groups, smartphone

## Abstract

**Background:**

Cancer survivors are at an elevated risk for several negative health outcomes, but physical activity (PA) can decrease those risks. Unfortunately, adherence to PA recommendations among survivors is low. Fitness mobile apps have been shown to facilitate the adoption of PA in the general population, but there are limited apps specifically designed for cancer survivors. This population has unique needs and barriers to PA, and most existing PA apps do not address these issues. Moreover, incorporating user preferences has been identified as an important priority for technology-based PA interventions, but at present there is limited literature that serves to establish these preferences in cancer survivors. This is especially problematic given the high cost of app development and because the majority of downloaded apps fail to engage users over the long term.

**Objective:**

The aim of this study was to take a qualitative approach to provide practical insight regarding this population’s preferences for the features and messages of an app to increase PA.

**Methods:**

A total of 35 cancer survivors each attended 2 focus groups; a moderator presented slide shows on potential app features and messages and asked open-ended questions to elicit participant preferences. All sessions were audio recorded and transcribed verbatim. Three reviewers independently conducted thematic content analysis on all transcripts, then organized and consolidated findings to identify salient themes.

**Results:**

Participants (mean age 63.7, SD 10.8, years) were mostly female (24/35, 69%) and mostly white (25/35, 71%). Participants generally had access to technology and were receptive to engaging with an app to increase PA. Themes identified included preferences for (1) a casual, concise, and positive tone, (2) tools for personal goal attainment, (3) a prescription for PA, and (4) an experience that is tailored to the user. Participants reported wanting extensive background data collection with low data entry burden and to have a trustworthy source translate their personal data into individualized PA recommendations. They expressed a desire for app functions that could facilitate goal achievement and articulated a preference for a more private social experience. Finally, results indicated that PA goals might be best established in the context of personally held priorities and values.

**Conclusions:**

Many of the desired features identified are compatible with both empirically supported methods of behavior change and the relative strengths of an app as a delivery vehicle for behavioral intervention. Participating cancer survivors’ preferences contrasted with many current standard practices for mobile app development, including value-based rather than numeric goals, private socialization in small groups rather than sharing with broader social networks, and interpretation of PA data rather than merely providing numerical data. Taken together, these insights may help increase the acceptability of theory-based mHealth PA interventions in cancer survivors.

## Introduction

Because of advances in early detection and treatment, the number of cancer survivors in the United States is increasing dramatically. In 2014 this number was an estimated 14.5 million, and by 2024 it is expected to increase to 19 million [1]. Despite advancements regarding cancer-related mortality, cancer survivors still face significant long-term health challenges, including an increased risk of all-cause mortality, obesity, type 2 diabetes, osteoporosis, anxiety, and depression [2]. Cancer survivors also face the risk of cancer recurrence and second cancers, sequelae like lymphedema and fatigue, and decreases in physical functioning that can impede the ability to conduct activities of daily living [2]. Physical activity for this population is generally safe and can play a vital role in ameliorating these physical and psychological challenges [2]. Despite this, most cancer survivors do not meet the minimum level of physical activity recommended by the American Cancer Society [3]. A study that interviewed a nationally representative sample found that only 30% of cancer survivors report meeting recommended levels of aerobic physical activity [4]. Innovative behavior change efforts are needed to increase physical activity in cancer survivors.

Mobile health (mHealth), utilizing mobile devices for health-related applications, has emerged as an important tool for health-related behavioral interventions [5]. The use of mobile devices has many potential advantages for such interventions, including the propensity for widespread dissemination, cost-effectiveness, the potential to minimize participant burden, sophisticated on-board sensors, the ability to provide immediate feedback, and the ability to provide experiences that are inherently enjoyable to users [6]. Importantly, cancer survivors are typically older adults, and technology use in this segment of the population is increasing rapidly [7]. Indeed, an increasing body of evidence indicates that technology-based interventions may be well received by cancer survivors and hold promise for physical activity promotion initiatives [8,9].

While there are many fitness and physical activity apps currently available for download, the majority of these apps are centered on measuring and improving athletic performance [10]. Such apps are generally not well suited for the majority of cancer survivors, who may be less motivated to engage in physical activity [11] and who face unique barriers to engaging in recommended levels of physical activity [12-14]. Using theory-based behavior change methods may be a particularly useful approach to increase physical activity in this population; however, most existing apps are not grounded in behavior change theory [15-17].

Incorporating users’ preferences has been identified as important for delivering technology-based physical activity promotion programs to older adults [18]. However, at present there is limited research to offer insight as to the practical preferences of cancer survivors for an app designed to increase physical activity levels. Puszkiewicz et al [19] conducted individual interviews with 11 cancer survivors regarding their experience with an existing physical activity app designed for the general population. Participants in this study reported that the app was generally well received but did not adequately address a number of factors relevant to understanding physical activity patterns in this population; these included fatigue, receipt of trusted information, cancer-related limitations, and social support. The authors of this study highlight the benefits of addressing such factors, as well as the utility such an app could provide as a means to facilitate physical activity–related communication between health care providers and cancer survivors.

Given the substantial resources required for software development, and the daunting reality that 23% of mobile apps are abandoned by the user after only one use [20], it is important to appropriately address the practical points regarding how an app may be well received and able to provide lasting value to the priority population. The purpose of this study was to use focus groups to generate insight as to cancer survivors’ preferences regarding the features and types of messages of an app to increase physical activity. Identified preferences were then applied to established behavior change methods [21-23], such as enactive mastery experiences [24] and verbal persuasion [25], to provide recommendations for future app development.

## Methods

### Recruitment

Inclusion criteria were that each participant be a survivor of stage I-III breast, colorectal, prostate, or endometrial cancer; be at least 18 years of age; have completed primary treatment; and have the ability to read and speak English. Participants were recruited (1) from survivorship clinics and support groups at MD Anderson (through a media-based approach that included distributing flyers, in-person presentations, and advertisements in MD Anderson’s internal and external publications), (2) in person at an MD Anderson Cancer Survivorship Conference, and (3) by sending a letter and placing a phone call to eligible individuals identified in the MD Anderson patient database.

### Focus Group Interviews

Data collection took place from November 2013 to March 2014. Each participant agreed to attend 2 focus group sessions at the MD Anderson Behavioral Research and Treatment Center and was compensated with a US $15 gift card at the completion of each session. Participants provided informed consent before the beginning of the study; this study was approved by the MD Anderson Institutional Review Board (protocol number 2013-0501). All focus group interviews were moderated by a master’s level senior research coordinator (female) with more than 3 years’ experience, trained in qualitative research methods; she conducted the interviews with the assistance of a semistructured interview guide and a colleague who took field notes (female, master’s level senior research coordinator with more than 10 years’ experience and trained in qualitative research methods). A demographic questionnaire, a measure of physical activity, and a questionnaire on technology use were administered at the beginning of the first focus group. Physical activity was assessed using a modified short-form version of the International Physical Activity Questionnaire (IPAQ-SF); this is a widely used measure with good test-retest reliability (.80) and acceptable criterion validity when compared with accelerometer data (median Spearman correlation =.30) [26].

**Figure 1:**
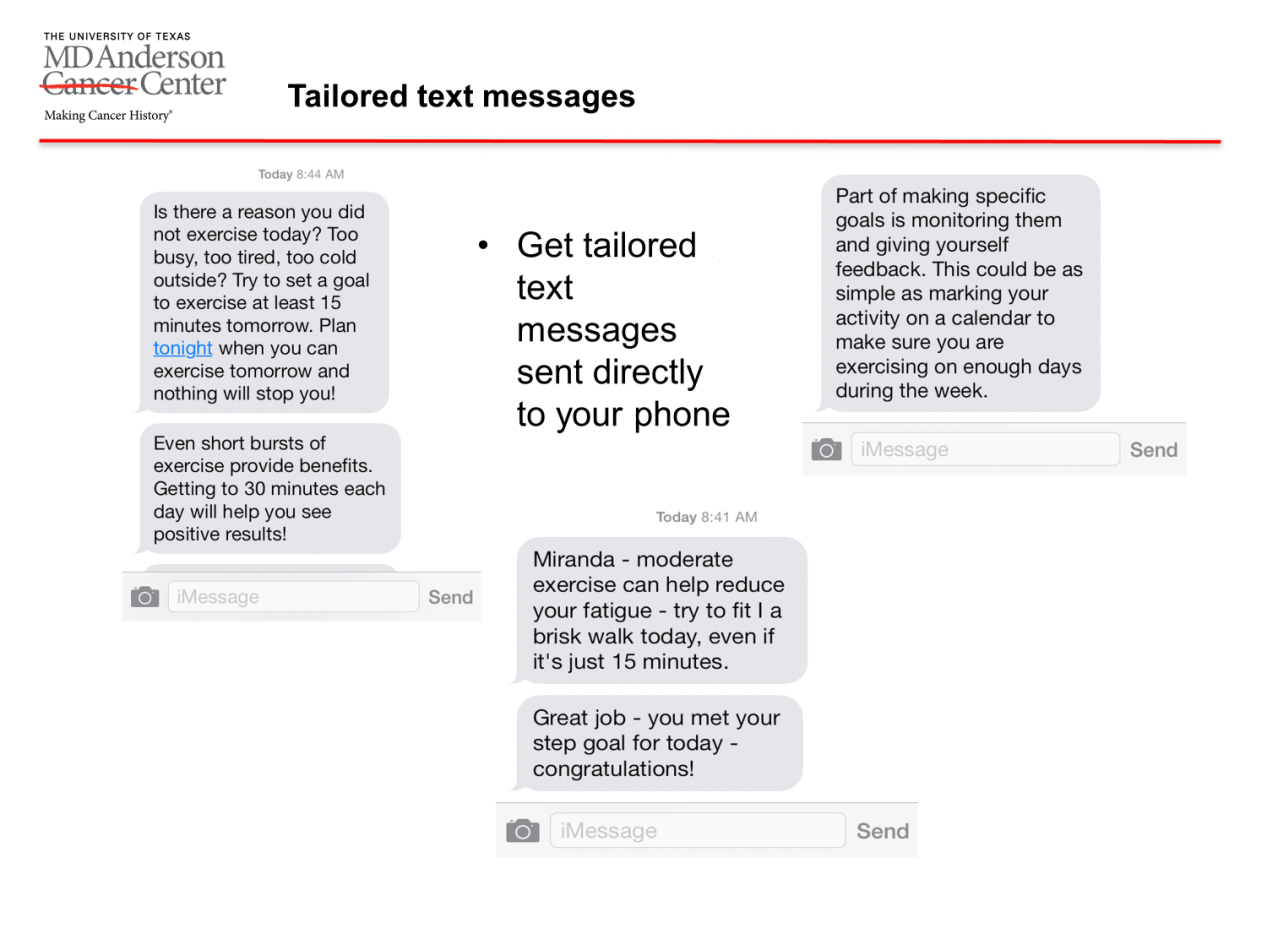
Example of a presented app feature: sending tailored text messages.

Each participant attended 2 focus groups, and each focus group consisted of 2 parts. An outline of the content covered is presented below (Multimedia Appendix 1). The first part of both focus groups was a discussion in which the moderator asked open-ended questions and followed with conversational probes as appropriate. These questions were derived from a combination of Social Cognitive Theory constructs (eg, goal setting) and practical questions (eg, texting preferences) [27]. The second part of both focus groups consisted of a slide show presentation, during which the moderator asked participants to share their thoughts and opinions on the featured content. In the first round of focus groups, the slide show featured various physical activity app features (Multimedia Appendices 2 and 3), such as receiving tailored text messages (Figure 1). In the second round of focus groups, the slide show featured 18 example text messages (Multimedia Appendices 4 and 5).

The researchers held weekly meetings in which they discussed the focus groups and reviewed field notes; additional focus groups were conducted until the researchers were confident that data saturation regarding the study’s research questions had been achieved. This was determined to be the case after a total of 13 focus groups had been conducted (8 focus groups in the first round were consolidated into 5 for the second round). Among the participants who attended the first focus group, 7 did not go on to attend the second. On average, each focus group had about 5 participants. All sessions were recorded with a digital audio recorder and professionally transcribed verbatim. Participants were asked to not use names during the focus groups, and surveys and transcripts were de-identified; all data were stored on encrypted, password-protected computers.

### Data Analysis

Transcripts were imported into the qualitative data analysis management program ATLAS.ti (version 7.0, Scientific Software Development GmbH, Berlin, Germany) [28]. Data analysis was conducted by 3 independent reviewers and consisted of both a deductive and an inductive phase. During the deductive phase, conducted first, for all transcripts 2 coders (ET and MCR) independently matched codes determined a priori to each comment that introduced a substantive point germane to this study’s topic. For the purposes of this study, this step served to allow the coders to become familiar with the data and screen out content that was not relevant. For the inductive phase, thematic content analysis was performed [29]. Codes were created and assigned to each discrete point made by each participant for all transcripts, and consolidated and organized in an iterative process to identify recurring themes and subthemes. A meeting (KMB-E, EJL, MLB, SS, and MCR) was held to resolve any differences in coding. Preliminary results were then presented to a third coder (SS), who verified the accuracy and exhaustiveness of the findings against all complete focus group transcripts. Finally, illustrative quotes for each subtheme were identified.

## Results

### Participant Characteristics

**Figure 2:**
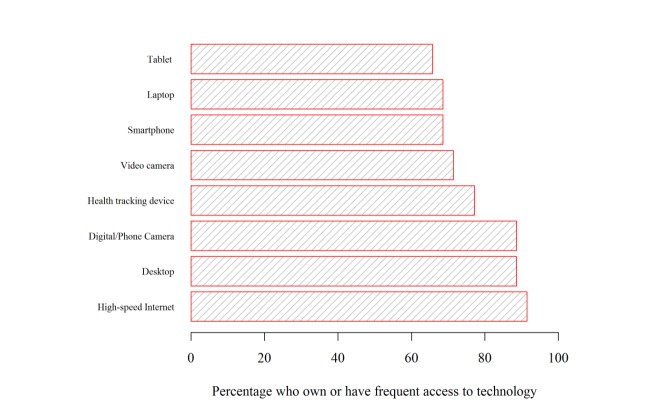
Access to technology.

The age of the 35 participants ranged from 41 to 84 years, with a mean of 63.7 (SD 10.8) years. Demographic characteristics are presented in Table 1. Participants were well educated, mostly female, and mostly white. Most (21/35, 60%) had been diagnosed with breast cancer. IPAQ-SF scores indicated that 41% (14/34) of participants did not meet recommended physical activity levels (Table 1). Most participants reported being very interested in technology (Table 1) and most participants (≥69%) reported having ready access to technological devices, a computer, and high-speed Internet (Figure 2).

**Table 1:** Participant characteristics.

Characteristic		n (%)
**Race**		
	Black	5 (14)
	Asian	4 (11)
	White	25 (71)
	Other	1 (3)
**Education**		
	≤High school	1 (3)
	Some college or 2-year degree	12 (34)
	Bachelor’s degree	7 (20)
	Master’s degree	12 (34)
	MD, PhD, or other advanced degree	3 (9)
**Cancer type^a^**		
	Breast	21 (60)
	Colorectal	4 (11)
	Endometrial	3 (9)
	Prostate	9 (26)
	Other	1 (3)
**Gender**		
	Male	11 (31)
	Female	24 (69)
**Current employment status^a^**		
	Employed full-time	11 (31)
	Employed part-time	1 (3)
	Not employed for pay, not seeking paid employment	5 (14)
	Not employed for pay but seeking paid employment	3 (9)
	Retired	18 (51)
	Homemaker	4 (11)
	Student	1 (3)
**Physical activity level (IPAQ-SF^b^** **)^c^**		
	High	5 (15)
	Moderate	15 (44)
	Low	14 (41)
**Interest in mobile and Internet technology**		
	Very interested	21 (60)
	Somewhat interested	13 (37)
	Not at all interested	1 (3)
**Self-reported skill with technology**		
	Very skillful or pretty skillful	14 (40)
	Somewhat skillful	17 (49)
	Not very or not at all skillful	4 (11)
**“I like to experiment with new technology”^d^**			
	Agree or strongly agree	24 (73)
	Neutral	8 (24)
	Disagree or strongly disagree	1 (3)

^a^Participants could indicate more than one option.

^b^IPAQ-SF: International Physical Activity Questionnaire–Short Form.

^c^Measure was completed by 97% (34/35) of participants.

^d^Item was completed by 94% (33/35) of participants.

### Themes

We identified 4 themes regarding participants’ preferences for an app to increase physical activity in cancer survivors: tone preferences, tools for personal goal attainment, prescription for physical activity, and a tailored experience. Subthemes within each overarching theme are presented along with illustrative quotes in Tables 2-5.

#### Tone Preferences

The first theme that arose was related to preferences for the tone of messages. Participants indicated that they would prefer messages to be casual, concise, and positive (Table 2). A casual tone was preferred to a clinical one; participants indicated that messages that are familiar, warm, friendly, and even funny would be more agreeable than those that were more formal. Proposed example messages were criticized as being too long, and participants frequently made comments explicitly stating that short messages are preferable to longer ones. Participants also indicated a preference for messages to exhibit a nurturing and supportive tone; they cautioned that, if not worded carefully, messages could be off-putting or even damaging. Messages that were perceived to be negative in any way were almost uniformly rejected. For example, a message that attempted to highlight the fact that walking is an excellent form of physical activity started with “While running or playing tennis might not be enjoyable...” and, as a result of this negative framing, was not well received. Finally, some participants expressed a preference for using a tablet to interface with an app. This was indicated as an easier way to access app content and manage frequent app messaging.

**Table 2:** Tone preferences.

Subtheme	Illustrative quotations
Casual	“I don't think you should be clinical with this, I think you should be funny, jovial, comical...something lighthearted to kind of boost your spirits up.” [P31]
	“Casual.” [P13]
	“Casual.” [P19]
	“I would do casual.” [P23]
Concise	“It’s like, God, if I see a message that long, I don’t know if I want to read it!” [P33]
	“Short, sweet. Remember, we don’t have a long attention span.” [P9]
	“The maximum length is a tweet.” [P8]
Positive	“They should sound positive...We’d want encouragement because every day we start over, and we need all the help we can get.” [P2]
	“I would love to get a little inspirational thing from some—especially when you’re in that position where you’re down. I like that.” [P31]
	“I mean, there was definitely a theme. We all like the positive versus the negative.” [P9]
Receptivity to using a tablet	“I use my iPad for texting and receiving. So I tend to look at it in the morning, midday, and evening. So I’m not constantly responding...I find it easier.” [P25]

#### Tool for Personal Goal Attainment

A second theme that was identified was that the app serve as a tool for personal physical activity–related goal attainment (Table 3). Participants indicated that physical activity goals tended to be manifestations of personally held values. For example, some participants wanted to be physically fit so as to be able to play with their grandchildren. Participants expressed a desire to be able to input personally held goals into the app, then be able to utilize the app as a tool for accomplishing them. Participants indicated they were more likely to engage in action planning if they had made a commitment to their peers, and the ability to use an app to enlist social support was identified as a noteworthy subtheme. Participants also talked about the potential utility of an app to provide periodic reminders to be physically active. They indicated that such periodic cues could help them to get on track for goal attainment, particularly if the reminder messages were delivered at opportune times. Such reminders were usually discussed in the context of a wearable device’s ability to detect prolonged bouts of inactivity and automatically send a cue to break up sedentary time.

Role model narratives emerged as a potentially powerful feature to empower cancer survivors to live a more physically active lifestyle. The notion of being presented with stories from individuals who had overcome salient obstacles was well received. One oft-cited caveat to featuring role model narratives was that such stories need to be relevant to the user; participants wanted to be matched to stories from individuals who had faced similar barriers. Were such matching not feasible, participants suggested allowing the user to individually browse for stories that they feel may be relevant to him or her. Participants cautioned against unwittingly presenting someone with the success story of a cancer survivor who was too physically active or did not face similar adversity.

Participants generally articulated a preference for either no social connection at all or a more private social experience that would allow them to enlist social support from those they know intimately and trust. Most participants said they did not want a social networking feature that would involve the public posting of information, such as one’s step count achievements or calories burned; instead, participants indicated that a social networking feature would be most attractive if such information were shared privately with a small group of user-selected friends or family. Using personal physical activity data as a means to compete with friends was not well received.

**Table 3:** Tools for personal goal attainment.

Subtheme	Illustrative quotations
Value-based goals	“One of my goals was to be able to keep being able to pick up my granddaughter, who is now five. And it was very important. And so now I’m using eight pound weights. I can still do it. And she’s big!” [P2]
	“Every time I exercise like I'm supposed to, I feel like there's a lot more likelihood that I'll live to see my grandkids married and all that.” [P5]
Ability to enlist social support	“When I’m with other cancer patients, I’m more motivated...So the exercise program would be tied into that support group...you know how you play Words with Friends and things like that?” [P7]
	“And that is what I need, is accountability to someone.” [P17]
Action planning for set goals	“Well, I’m more successful when I have it scheduled in my life...I definitely need that structure.” [P9]
	“So that would be an excellent message to say, ‘In your goal planning, plan a plan B and a plan C if plan A doesn’t work out for that day.’” [P34]
Reminders from app	“I think just that a reminder like...sending some kind of a text of, ‘Here’s some exercises to do for today. See if you can do this 10-minute deal.’” [P7]
	“Remind me that I had to do it. It would be the last little kick when I'm sitting there getting ready to pour the next glass of wine, or the first glass of wine before dinner.” [P8]
	“Just saying, you know, ‘What’s going on? I’ve noticed that you’ve not been logging on. Is there a problem?’ or, ‘Is there some way we can help you get back on track?’” [P15]
Role model narratives	“I think that’s very encouraging because each and every patient has their own story and how and why they have cancer, and how they can succeed and move on and live a healthy life.” [P18]
	“It’s always nice to hear about people who have done it and how they struggled and how they overcame their struggle to get to their successful point.” [P6]
	“Stories that you can opt in or out of or read or not read...so you read a story and go, ‘Oh, that’s really nice, except it doesn’t apply to me.’” [P34]
Social networking	“No. That's too personal. I don't post anything personal, really.” [P26]
	“I agree, I mean I don't like just to put progress on my weight or whatever to everybody, all my friends or whoever. But if there is some group...” [P4]
	“No. I don't like to compete with anybody. I mean I like to compete. But I'm always competing against myself. And to put it next to somebody else, that would defeat me.” [P17]

#### Prescription for Physical Activity

Another overarching theme concerned the presentation of a prescription for healthy types and levels of physical activity for cancer survivors (Table 4). Participants indicated a desire to be presented with concrete, short-term goals for physical activity that would ultimately help them realize their more abstract, value-based goals. Participants stressed the importance that recommended goals be attainable and come from a trusted source (eg, their cancer hospital or an authoritative health agency). Furthermore, participants reported wanting app features that could help the users appreciate progress and visualize incremental improvements related to their recommended goals.

Participants also expressed a desire to be presented with new ways of being physically active and to be educated about how to perform new exercises safely. To this end, participants expressed a strong preference for video demonstrations over text or pictures. Participants also indicated a desire for receiving a summary of the relevant literature on physical activity and cancer survivorship presented in layman’s terms; they noted that such information could be very motivating and that some confusion exists over what is perceived to be inconsistent recommendations for physical activity in cancer survivors. Many participants voiced surprise at the fact that physical activity can reduce the risk of cancer recurrence for some types of cancer. Again, an important qualifier identified for receiving this kind of information was that it come from a reputable and trusted source.

**Table 4:** Prescription for physical activity.

Subtheme	Illustrative quotations
Goal suggestions	“If you’re a runner or a runner wannabe...then maybe in your app you say, ‘What kind of activities are you doing or you want to be doing?’...your suggestion could be like...‘Have you been thinking about doing a 5k?’ or, ‘If you want to do a 5k, here’s what you need to do.’” [P33]
	“So if this machine knows that you’ve been sitting, maybe it can suggest some exercises you can do when you’re sitting, or mention that you’ve been sitting for an hour, and after an hour you should get up and just walk around for ten minutes or something like that.” [P6]
Novel physical activity suggestions	“Or tightening your stomach while you’re sitting in a chair in the office kind of thing, or standing instead of sitting when you're doing an activity.” [P7]
	“Or something like, ‘Do you know there's free programs at the park?’ or something, or like, ‘Do you know most gyms you can get free membership to try out different classes?’ Stuff like that.” [P33]
Physical activity demonstration	“So have a video that says ‘here’s how you do a squat.’ If you’re not able, ‘here,’ it shows you how to do a modified squat...or, ‘here’s how you get up out of your chair and do a stretch: here’s the modified stretch, here’s the full stretch,’ because people are at different levels of ability.” [P6]
	“I think video is helpful just for demonstrating the whole thing for somebody who may not have ever done it before, it’d be good to see.” [P7]
Digest of research literature	“Information, though, about—true information about, let’s say something new came out that if you do X, X number of times a day, your risk of whatever would go down by... ‘Research has shown that...’ that would probably motivate me more than a reward or punishment.” [P15]
	“I would like some research that's been done. So what do we need to do? What does research show, generally speaking? What do we need to do before we get started?” [P5]

#### Tailored Experience

A final theme identified from the focus group interviews was a preference that an app provide an experience that is highly tailored to the individual user (Table 5). This emerged as an important parameter of use for many of the subthemes presented above. Frequently cited factors to incorporate for individualization included cancer-related information, age, personal health concerns, physical limitations, physical activity preferences, location, weather, current physical activity levels, and trends over time.

Participants indicated wanting to be recognized and congratulated for activity-related achievements and presented with information about how such achievements translate into physiological processes (eg, calories burned). They talked about wanting to be able to see and track changes in activity levels over time, along with corresponding changes in health indicators (such as waist measurement, body mass index, cholesterol, blood pressure, and heart rate). This was often discussed in the context of incorporating a wearable fitness tracker. Participants emphasized a strong preference for information to flow from the app to the user and not the other way around. They stressed that a burdensome process of inputting data would pose a great threat to sustained use of the app. Participants reported wanting rich, personalized data, especially to inform such features as physical activity goal suggestions and personalized role model narratives. Generally, suggested weekly step count goals should be based on an incremental increase from the user’s previous week’s step count, and role model narratives from especially active individuals should not be presented to individuals who are less active.

Participants expressed the importance of receiving information that is relevant for their unique health profile; they suggested that the app offer content that is sensitive to user-identified information, such as cancer diagnosis, personal health considerations, age, and physical limitations. For example, they reported wanting exercise demonstrations that are sensitive to the user’s physical limitations and novel ways to perform physical activity that would not aggravate such limitations. Also, participants expressed a preference to be able to interface with the app to indicate health-related changes. If, for example, an injury were to occur, participants indicated that they would like to be able to note this in the app and receive a temporary reduction in message frequency or altered message content.

Finally, participants made suggestions for tailoring content based on the user’s location. Participants appreciated the idea of being presented with nearby opportunities to engage in physical activity. Walking paths, public parks, outdoor events, and yoga or Pilates studios were identified as some opportunities that an app might inform the user of. Poor weather was repeatedly cited as a barrier for engaging in physical activity, and it was suggested that the app might address this by providing recommendations for alternative activities if this was the case.

**Table 5:** Tailored experience.

Subtheme	Illustrative quotations
Extensive yet passive data collection	“I don't want an app...where you have to record every ounce of food that you eat. I've tried one of those before, and they're really painful to use, actually, where you record everything that you eat and everything you do. I just want an app that records how much I exercise and when I exercise and the results of that exercise, how much-what my weight is, and maybe what my waist measurement might be.” [P8]
Recognition of physical activity	“I think it is important too to get acknowledged on a daily basis.” [P7]
	“Something, that if you achieved a particular goal, it would be great to get a text message saying you’ve reached your goal. Or that you’ve taken 10,000 steps daily for the last six weeks or whatever it is.” [P8]
Individualized data about progress and biological processes	“How much that you did...So then you’d be able to go back historically and look by the week or by the month at what you're able to do. And then feel good about what you did, or maybe not so good.” [P17]
	“It would also be useful...(to) keep track of things like (heart rate) blood pressure and cholesterol and BMI, waist measurement.” [P8]
Input personal health concerns	“I think you have to think about physical restrictions. Some people have back issues. You know, some people have knee issues, shoulders. And I don’t know if there’s a way to individualize that so you can build that in for each individual with a questionnaire, perhaps, before you start.” [P26]
	“So like I said, there may just be categories by age or by limitation, because there could be a juvenile person who had leg cancer. I mean that’s a possibility. So you pick the category that best fits you or best describes your limitations. And then maybe the exercises or the suggestions are focused on that. And I agree with you. Most of us probably are 30 and older.” [P19]
Personalized role model narratives	“So more about overcoming some barriers with it and feeling the success stories about how it worked for one person that might not necessarily apply to us because—one of the focus groups I was in, there was a lady who’s a runner. I mean she’s running miles and miles and it’s like, ‘Ugh.’ Her situation doesn’t apply to me personally.” [P34]
	“And whatever other cancers that there might be, I think you should have it specific for them and say, ‘Okay, this is what I did because I was going through this. And this is how I felt when I went through this.’” [P31]
Nearby physical activity resources based on the user’s location	“So if you have it location-specific, where the patients are and what's available in their neighborhoods and their areas, it's not within a five-mile or a twelve-mile radius of them, that is something that they can go to.” [P31]
	“Or there is free yoga classes out in the park or like Discovery Green or something. Then you don't have to pay and you can go try it. You don't have to really sign up with a yoga studio or something. So there are a lot of options out there.” [P25]

## Discussion

### Principal Findings

In this study, we conducted focus groups to ascertain cancer survivors’ preferences for the features and types of messages of an app to increase physical activity. We identified 4 overarching themes for desired app content: (1) clear, positive, and concise messages, (2) various tools for personal goal attainment, (3) an appropriate prescription for physical activity, and (4) an experience that is tailored to the individual. Taken together, our results indicate that participants want an informal interface with an app that provides a highly individualized experience to facilitate engagement in healthy levels of physical activity. This can be achieved by an app that provides real-time feedback and personalized content sensitive to the user’s unique health concerns and physical activity preferences.

### Comparison With Prior Work

In their study, Puszkiewicz and colleagues [19] conducted in-depth interviews and used thematic analysis to identify themes related to cancer survivors’ feedback on an app designed to increase physical activity. The 4 themes identified in this study included (1) barriers to physical activity, (2) receiving advice about physical activity from a reliable source, (3) tailoring the app to one’s lifestyle, and (4) receiving social support from others. Our study complements these findings. Similarities include the importance of the perceived trustworthiness of a physical activity app and the ability of the app to provide tailored content to the user. Puszkiewicz and colleagues also identified a preference for receiving social support from others. Results from our study qualify this finding by highlighting privacy concerns; one way to address this would be to avoid public social network postings in favor of more carefully matched, private connections. Puszkiewicz and colleagues also point out the potential utility a physical activity app may have for health care providers, who often are unable to adequately discuss physical activity with patients owing to competing demands for time.

In accordance with the findings of this study, in a review of the literature Higgins [30] found that tailored physical activity feedback is associated with apps that are more effective at inducing behavior change, and that decreasing participant burden tends to increase adherence rates. However, qualitative work done by Miyamoto et al [31] found that simply tracking and presenting data may not be sufficient to lead to long-term behavior change maintenance, and that the context of this feedback is critical. Findings from our study provide insight on some contextual issues that may improve acceptability and, ultimately, efficacy of such apps (eg, presenting physical activity feedback alongside the implications of meeting recommended physical activity levels on one’s risk of cancer recurrence, or personal health concerns such as lymphedema).

Results of this study are consistent with previous research findings for the preferences of a physical activity app in the general adult population. Similarities found by Dennison and colleagues [32] include preferences for minimal user burden, backing by a trusted source (eg, hospital), inclusion of a goal setting and monitoring component, feedback and advice on how to change behavior, accurate information and tracking features, messages that have a positive tone and are not too frequent, and privacy protection. In formative research for the development of an app to increase physical activity in the sedentary adult population, Rabin and Bock [33] identified participant preferences that included automatic tracking of steps, feedback on physical activity accomplishments, goal setting, and suggestions for how to overcome barriers. Dennison and colleagues [32] found some additional preferences not directly identified in our study: participants expressed a desire for an app that is free, can be easily turned off, does not negatively affect other device uses, has clarity about what it will do, and does not present undue surprises. These additional findings may hold true for cancer survivors.

In their formative development of iCanFit, a Web-based app to increase physical activity in older cancer survivors, Hong and colleagues [34] presented 6 key functions. These were “Locator, Goals, Community, Healthy Tips, Library, and Support” features, which served to provide a tailored experience regarding local resources for physical activity, the ability to input short-term and long-term goals, social networking features, advice providing a prescription for healthy living, access to relevant literature, and technical support, respectively. These features are concordant with the findings of our study. Technical support was not an explicitly identified theme in our study but may be particularly important given that older cancer survivors may not be as tech-savvy as the general population; indeed, this study found that most (21/35, 60%) rated themselves as somewhat, not very, or not at all skillful with technology.

Cancer survivors are generally older adults, so an app to increase physical activity in this population may face challenges due to lower rates of technology use in this population. A study conducted by Martin and colleagues [35] found that cancer survivors’ interest in interventions delivered by a mobile phone was relatively low. However, this study used data from 2010, and older adults’ use of technology is increasing rapidly [7]. Part of a reported lack of interest of mobile phone use in this population may be due to age-related declines in vision and manual dexterity. Martin and colleagues did find a relatively high interest in older adults for interventions delivered via computer, but they did not explore cancer survivors’ perceptions and interest in tablets. The use of tablets may circumvent some of the physical challenges faced by older adults due to having larger screens that offer higher visibility and a larger touch screen. There also may be a difference in perception: some evidence indicates that older adults may tend to view smartphones as especially complex phones, while on the other hand viewing tablets as relatively simple computers [36]. Indeed, several comments made by participants in this study corroborate this notion, and a recent survey showed that tablet and e-book reader ownership in older adults is higher than smartphone ownership [7]. Future studies should explore cancer survivors’ interest in this intervention modality.

### Implications for Research and Practice

Our findings suggest that presenting goal-setting exercises in the context of participants’ personally held priorities and values may be a particularly useful approach to elicit intrinsically motivating goals. Self-determination theory posits that greater internalization of goals is more likely to lead to lasting behavior change [37], and empirical tests in physical activity support this notion [38]. This may be accomplished by a program that has the users reflect on and identify their values and then generate physical activity–related goals in light of this content; this input could then be periodically leveraged in order to maximize participants’ physical activity adherence. An app may be especially well suited for this owing to onboard technological components, such as a camera that could capture values (eg, pictures of grandchildren), and an onboard accelerometer or the ability to sync to wearable activity tracking sensors that could responsively register changes in physical activity levels.

Importantly, many of the desired features articulated are compatible with both empirically supported methods of behavior change and the relative strengths of an app as a delivery vehicle for behavioral intervention [21-23]. For example, participants talked about being presented with stories from other cancer survivors who have overcome similar obstacles and also being presented with instructional videos demonstrating how to perform various physical exercises. These preferences align well with behavioral change methods (behavioral journalism and demonstration of behavior, respectively) theorized by Bandura’s Social Cognitive Theory to influence behavior via observational learning [27]. Table 6 presents our suggestions for how an app might incorporate behavior change methods. We arrived upon these suggestions by applying the preferences identified in this study to empirically supported behavior change methods drawn from both the Intervention Mapping approach [21,22] and Michie and colleagues’ [23] Behavior Change Technique Taxonomy.

Future formative research for the development of an app to increase physical activity levels in this population might corroborate these findings with quantitative data and provide insight as to the relative rank-ordered preferences of desired app features and messages. It would also be useful to ascertain what qualities of a physical activity–related app are associated with higher rates of participant engagement (eg, messaging or notification frequency, type of content featured, social networking features). Additional studies are needed to determine whether an app-delivered intervention can lead to increased physical activity initiation and maintenance in cancer survivors and, if so, which behavior change methods might be the mechanisms through which these outcomes are achieved.

**Table 6:** Behavior change recommendations that may improve acceptability.

Behavior change methods [21-23]	Recommendations
Enactive mastery experiences [24]; set tasks on a gradient of difficulty [25]	Have the user start with a physical activity–related goal (eg, step count) that is comfortably accomplished and have goals incrementally increase over time
Consciousness raising [39]	Dispel commonly held misconception regarding barriers to physical activity by offering a digest of relevant literature (eg, address the misconception that physical activity is contraindicated if one is at risk for lymphedema)
Goal setting [40]; self and environmental reevaluation [39]	Encourage users to reflect on personal values during goal setting and the potential outcomes of behavior change from multiple perspectives; encourage users to create value-based goals for physical activity
Tailoring [39,41]	Maximize mHealth program potential to provide specific, personalized information relevant to the user; minimize participant data entry burden
Self-monitoring or feedback on behavior [42]	Go beyond simply presenting physical activity summary information; provide interpretation of personal physical activity data relevant to users’ health concerns and cancer experience
Stimulate communication to mobilize social support [43]	Feature private sharing outlet with personal friends and family, or match user to others who have experienced a similar cancer journey; avoid sharing indiscriminately to broader social network
Behavioral journalism [25,44]	Offer role model narratives that demonstrate that others, like the user, can overcome salient barriers and experience real benefits regarding physical activity
Guided practice [23]	Provide videos for recommended exercises that demonstrate proper technique and address personal physical limitations and health concerns; provide individualized feedback regarding user’s performance
Providing cues to action [45]	Offer periodic prompts to influence behavior by making it more salient in the mind of the user; allow the frequency of messaging to be determined by the user to minimize perceived burden
Verbal persuasion about capability; improving physical and emotional states [25]	Assume a casual tone from a trusted source; provide positive reinforcement by celebrating successes, and provide minimal negative content

### Strengths

A strength of this study’s focus group qualitative approach is the ability to generate rich data to provide insight that extended beyond the preconceived notions of the researchers. This study’s use of 3 coders to analyze the data in a systematic, iterative process was a strength, as was the use of 2 phases of data analysis to strengthen the authors’ familiarity and understanding of the content.

### Limitations

A potential limitation of this study includes the use of recruitment methods that may have introduced self-selection bias; individuals who agreed to participate may have been especially active or interested in technology. However, results indicate that this threat may not be particularly salient, as IPAQ-SF scores categorized nearly 42% (14/34) of participants as exhibiting “low” physical activity levels. Still, our sample’s educational level and racial/ethnic diversity does not match that of the larger priority population, which may limit the generalizability of our findings. Furthermore, the generalizability of our findings is limited by the fact that the majority of participants were female, breast cancer survivors. Different types of cancer can lead to unique patient experiences regarding physical limitation and psychological challenges [46]. For example, breast cancer survivors may be more likely to suffer from depression than lung cancer survivors but less likely than those diagnosed with brain cancer or females diagnosed with genital cancers [47]. Preferences for forms of mobile or Web-based support may also differ across cancer types, possibly owing to these different experiences [35,48]. Indeed, quantitative and qualitative studies of individuals with different cancer types have found different experiences and different preferences for online support [49-51]. Accordingly, our findings may not be applicable for survivors of certain types of cancer. Another limitation was that the focus groups were not homogeneous with respect to participants’ physical activity level. This may have had the effect of systematically influencing the dynamic of the sessions and created a bias in the data. While qualitative research methods can be especially effective at generating a comprehensive breadth of information on a particular topic, as they were conducted here, little insight was provided on the relative rank of preferences for the many app features identified. Given the resources required for app development generally, and the inherent challenge of providing an app that is able to satisfy all identified preferences, narrowing this list in order to focus on priority app features may be necessary.

### Conclusions

Given the dramatic uptake in technology use, utilizing an app as a modality for behavioral intervention holds promise for increasing physical activity in cancer survivors. Presenting rich physical activity data and feedback, while minimizing user data entry burden, would be a critical feature of such an app. Results of this study provide preferences that may be used to enrich the context in which an app provides physical activity feedback. Useful approaches may be to capitalize on personally held values during the goal-setting process, to present an individualized prescription for physical activity from a trusted source, and to provide tools that facilitate goal fulfillment. Future studies should incorporate the perspectives of oncologists and other health care providers, as well as test these findings in a pilot version of an app to increase physical activity in cancer survivors.

## Appendix

Multimedia Appendix 1 Content covered in focus group sessions.

Multimedia Appendix 2 Slideshow of various app features.

Multimedia Appendix 3 Percentage of positive feedback for various app features.

Multimedia Appendix 4 Slideshow of example text messages.

Multimedia Appendix 5 Ranking by percentage of positive feedback for specific messages.

## Abbreviations

IPAQ-SF: International Physical Activity Questionnaire–Short Form
