# Supplementary material for: Mobile Health Physical Activity Intervention Preferences in Cancer Survivors: A Qualitative Study
Source: JMIR Mhealth Uhealth. 2017 Jan 24;5(1):e3. doi: 10.2196/mhealth.6970 (PMC5296620; doi:10.2196/mhealth.6970)
Supplement: Multimedia Appendix 1 [file mhealth_v5i1e3_app1.pdf]

| Focus Group Session 1 | Focus Group Session 2 |
|---|---|
| Part 1: Discussion Questions on Exercise Habits and Preferences:<br><br>How do you feel about your current exercise habits?<br><br>Have you had difficulty maintaining an exercise program? What were the problems?<br><br>What information do you need about exercise?<br><br>What helped you/would help you get started with an exercise program?<br><br>What helps you/would help you stick with an exercise program?<br><br>How would a mobile application and SMS-based program address your exercise concerns?<br><br>What features would be most helpful and would need to be included for it to be used by you?<br><br>From our list of features, which of these two is more important? (Continue through list)<br><br>Would you want to join a program like this? Why or why not?<br><br>Have you used a smart phone application to help you start or maintain an exercise program? What was it? What features of that program did you find useful, and why? Which were not useful, and why? | Part 1: Discussion Questions for Text Message Preferences and Goal Setting Behaviors:<br><br>Do you have any rules for texting?<br><br>Do you use abbreviations when you text?<br><br>For texts that would be coming from us, would you prefer they be casual as if a friend wrote them or more formal as if a doctor sent them?<br><br>What tone would you prefer in your texts?<br><br>What types of messages would increase your confidence to exercise? *(Self-efficacy)*<br><br>What can the texts focus on in order to increase motivation? *(Observational Learning-Motivation)*<br><br>How should we address messages that are about regulating exercise through goal setting or self-monitoring? *(Self-Regulation)*<br><br>What types of feedback would you be interested in receiving via text? *(Self-Regulation)*<br><br>Would you enjoy hearing brief stories about a cancer survivor peer who has successfully started exercising or who is about to start one? *(Social Modeling)*<br><br>Would you like receiving messages with specific instructions a brief exercise that you could do at that moment? Why or why not? *(Mastery Experience)*<br><br>How do you feel about receiving weekly tips or quizzes followed up with correct answers? (*Knowledge)*<br><br>How do you go about setting exercise goals? What kind of goals do you set?<br><br>How do you react when you do or don't reach your goals? |

| | |
|---|---|
| How often do you think that you would use this program? How often would you want to receive text messages?<br><br>What are some of the barriers to joining a program such as this? | |
| Part 2: Slideshow Presentation: 13 Exercise App Features<br><br>Exercise by Muscle Group (Get exercise ideas for the specific muscle you want to target, Track your progress over time, Detailed instructions on how to use gym equipment)<br><br>Electronic Tracking of Steps (Track your steps throughout the day, Track your steps, distance, floors, and calories electronically)<br><br>Tailored Text Messages (Get tailored text messages sent directly to your phone)<br><br>Exercise Instruction (Step-by-step instructions and video demonstrations)<br><br>GPS Tracker (View the route of your run/walk; Track distance, pace, and duration)<br><br>Measurement Tracker (Track your measurements over time, Track hip, waist, and neck circumference)<br><br>Fitness Routine Tracker (Track your exercise, Group exercises into separate workouts)<br><br>Earn Medals (Earn medals for different activities you accomplish)<br><br>Distance Training (Audio coach tells you when to walk or run, Listen to your music) | Part 2: Slideshow Presentation: 18 Example Text Messages<br><br>Excellent job this week – you met your step goal on 5 out of 7 days!<br><br>Choose a few people who are as committed as you to being active and who live or work where it is convenient for both of you.<br><br>Exercising is way more enjoyable when motivation can flow both ways.<br><br>Keeping track of your exercise and looking at changes over time can help you stick with the program! Don't forget to wear you pedometer and check your progress in the app!<br><br>Only 820 more steps to go until you meet today's goal – good work!<br><br>Does a 10 minute workout sound better than a 30 minute workout? Many studies have shown that doing 3 10-minute bouts of exercise provides the same benefits as 30-minutes all at once.<br><br>You are amazing! By exercising over 45 minutes you are taking very proactive steps to lower your chance of cancer relapse. It is very important though to ensure that you are getting adequate warm up and cool down periods to prevent injury.<br><br>Setting a realistic exercise goal and then working to achieve it can help keep you on track. What is your exercise goal this week?<br><br>It is easy to find reasons to not exercise, but exercise is not an "all or nothing" activity. If you skip a few days (or even weeks) start small and take brisk walks during your break and lunch times. It all counts toward your daily 30 minutes.<br><br>It is great that you are confident in yourself and your ability to exercise! Did you know that regular moderate exercise helps improve daily sleep and prevent osteoporosis?<br><br>So you say you don't like exercising…try pairing it with something you do like – watching a good TV show while walking on the treadmill or listening to music while doing resistance exercise.<br><br>You made the daily recommended amount! Congratulations! Take a moment to reflect and remember how it was possible to work out this long today. With a plan in place it will be easier to keep incorporating exercise as part of your daily routine! |

| | |
|---|---|
| Competing with Friends (Connect with friends and compete)<br><br>Muscle Map (Keep track of specific muscle progress over time)<br><br>Connect with Social Media (Post your progress on Twitter or Facebook) | If you’re having trouble sticking with an exercise program, think about what has helped you exercise or change any habit in the past – can it help you now?<br><br>More and more cancer survivors are stepping up to exercise – how about you?<br><br>You are in a good place! Exercise is a priority for you so don't let surprises during the day keep you from exercising!<br><br>How will you reward yourself for meeting your exercise goals today?<br><br>It’s normal to feel a bit tired after exercising – it should get better with time. If you feel completely exhausted after exercise it might be good to exercise for less time or with less intensity for a few days.<br><br>Running or playing tennis may not be enjoyable to you, but remember that walking is an excellent form of moderate intensity exercise. It is easy, convenient, inexpensive, and can be social!<br><br>Exercising can help increase your daily metabolism and help you maintain your weight long-term. Try purchasing an exercise magazine the next time you are at the grocery store and see if there are any new tips for you to use! |
