# Supplementary material for: Mobile Health Physical Activity Intervention Preferences in Cancer Survivors: A Qualitative Study
Source: JMIR Mhealth Uhealth. 2017 Jan 24;5(1):e3. doi: 10.2196/mhealth.6970 (PMC5296620; doi:10.2196/mhealth.6970)
Supplement: Multimedia Appendix 2 [file mhealth_v5i1e3_app2.pptx]

## Slide 1
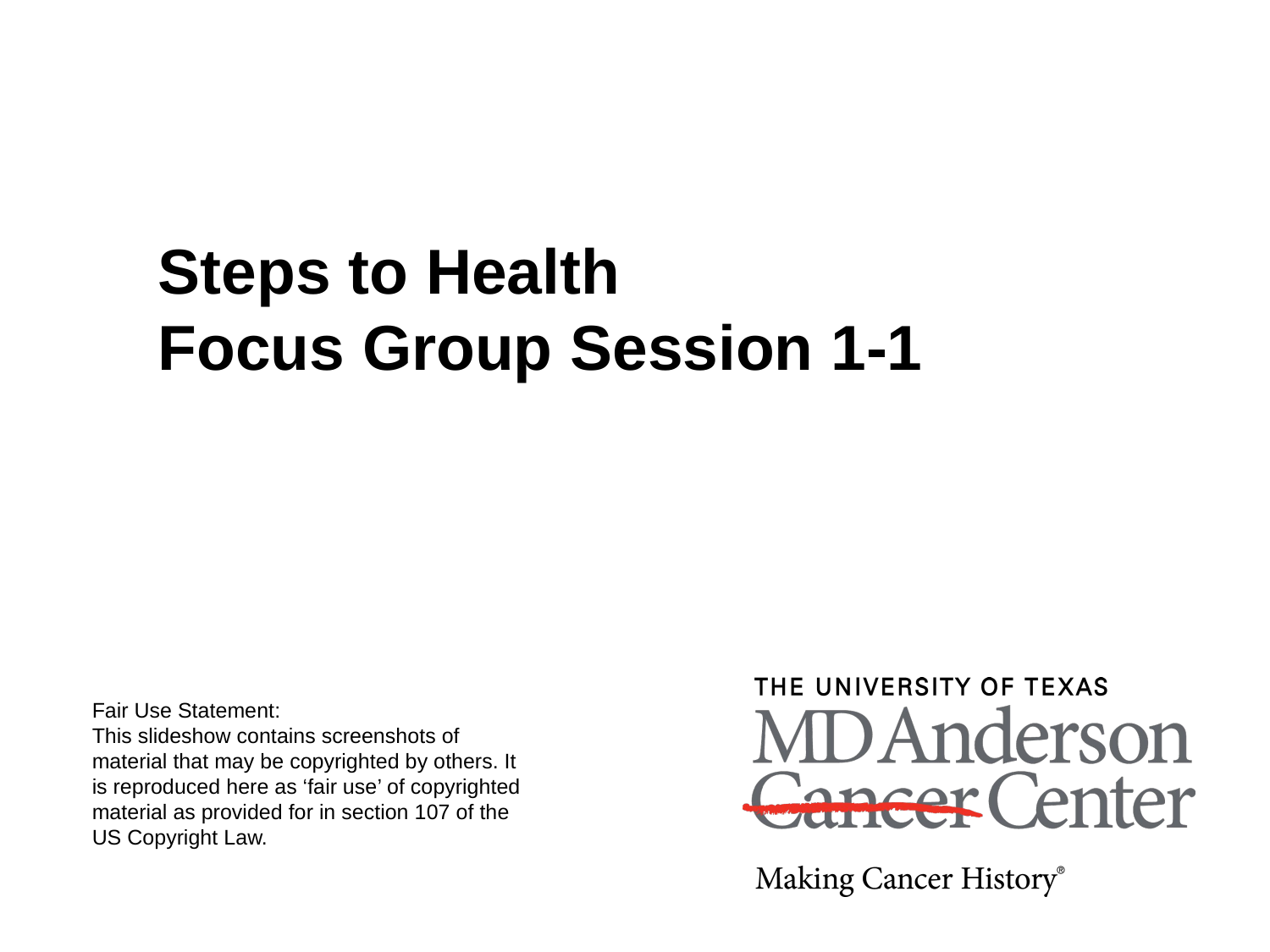

Steps to Health
Focus Group Session 1-1
Fair Use Statement:
This slideshow contains screenshots of material that may be copyrighted by others. It is reproduced here as ‘fair use’ of copyrighted material as provided for in section 107 of the US Copyright Law.

## Slide 2
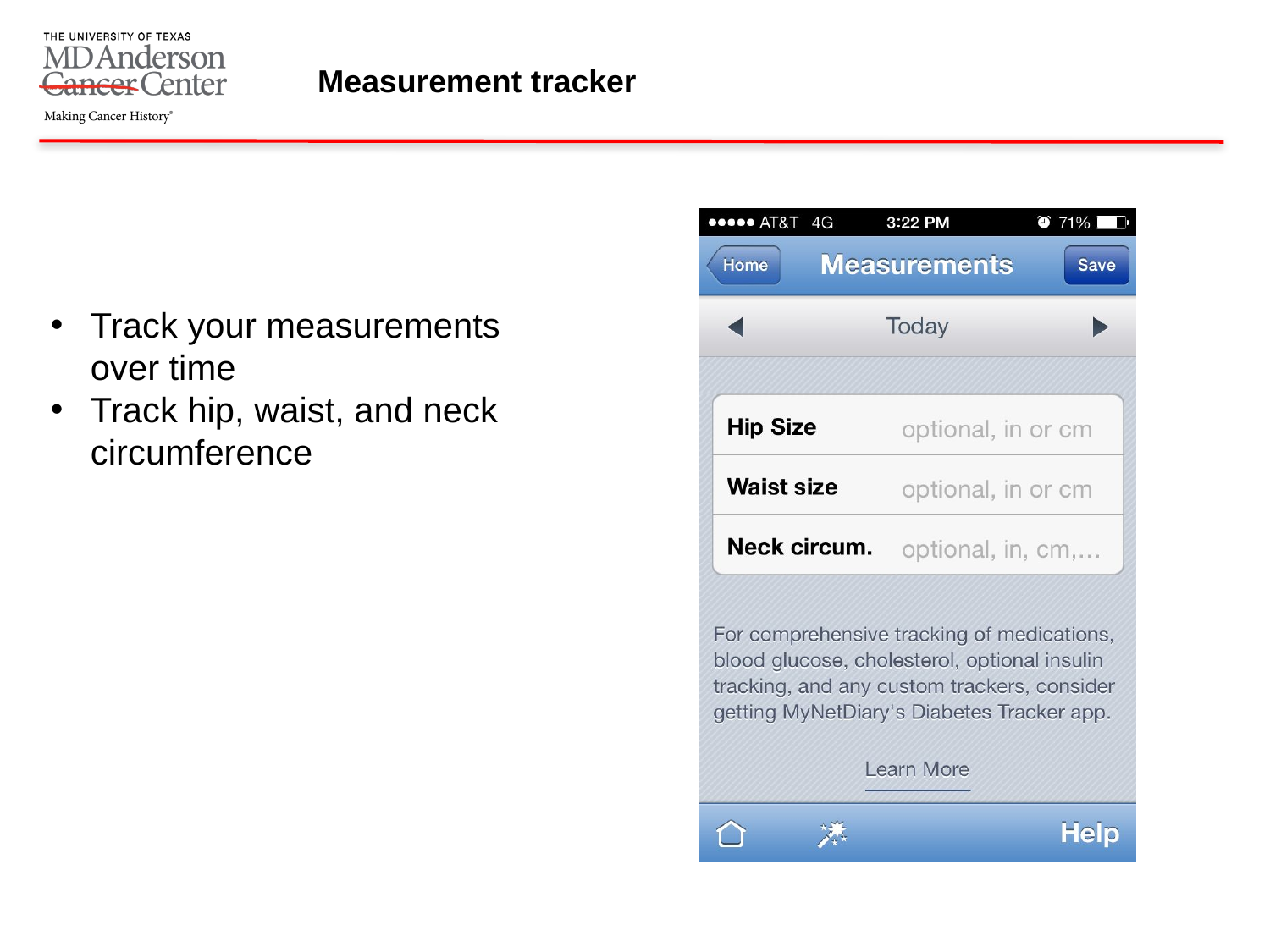

Measurement tracker
Track your measurements over time
Track hip, waist, and neck circumference

## Slide 3
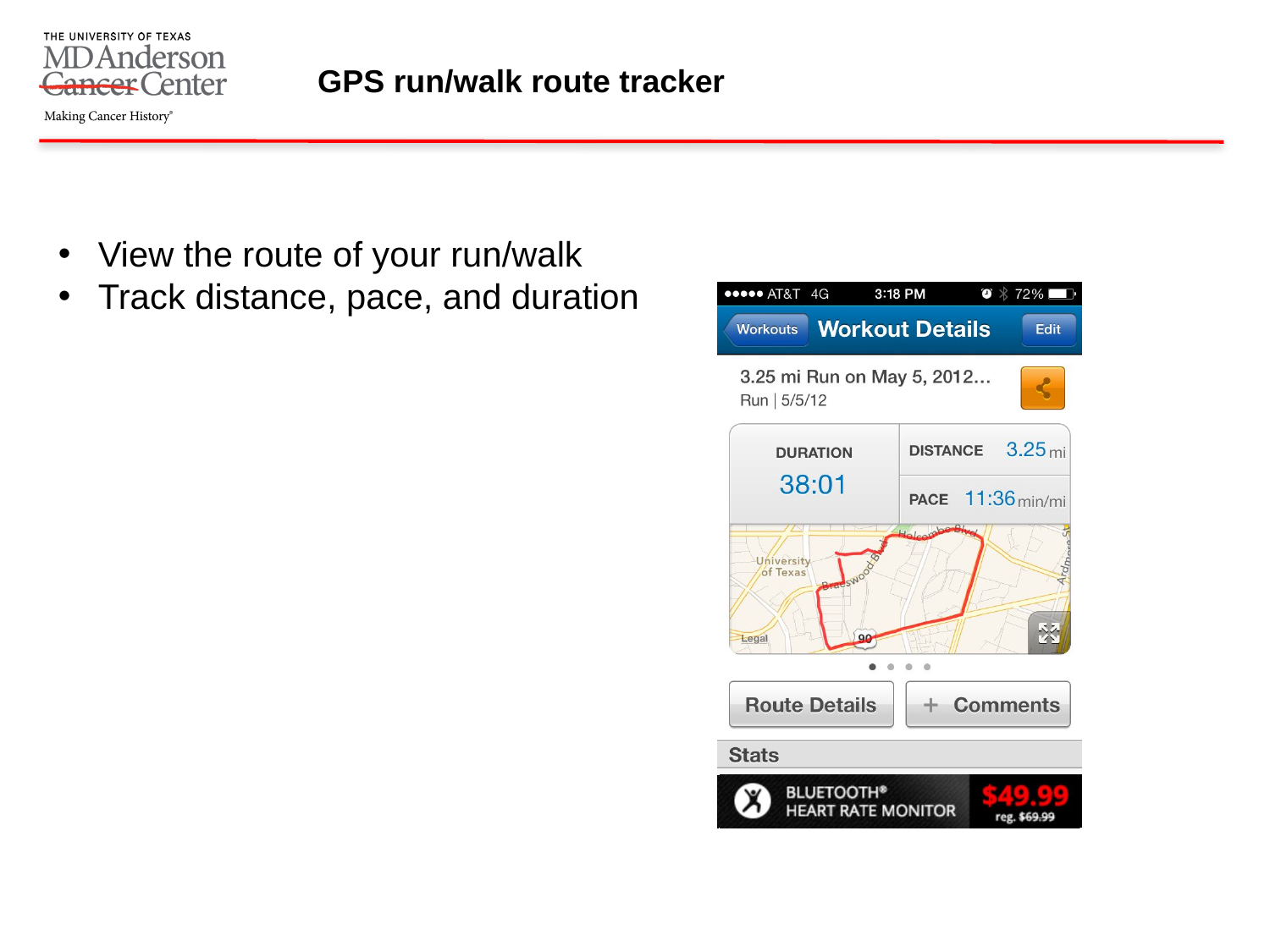

GPS run/walk route tracker
View the route of your run/walk
Track distance, pace, and duration

## Slide 4
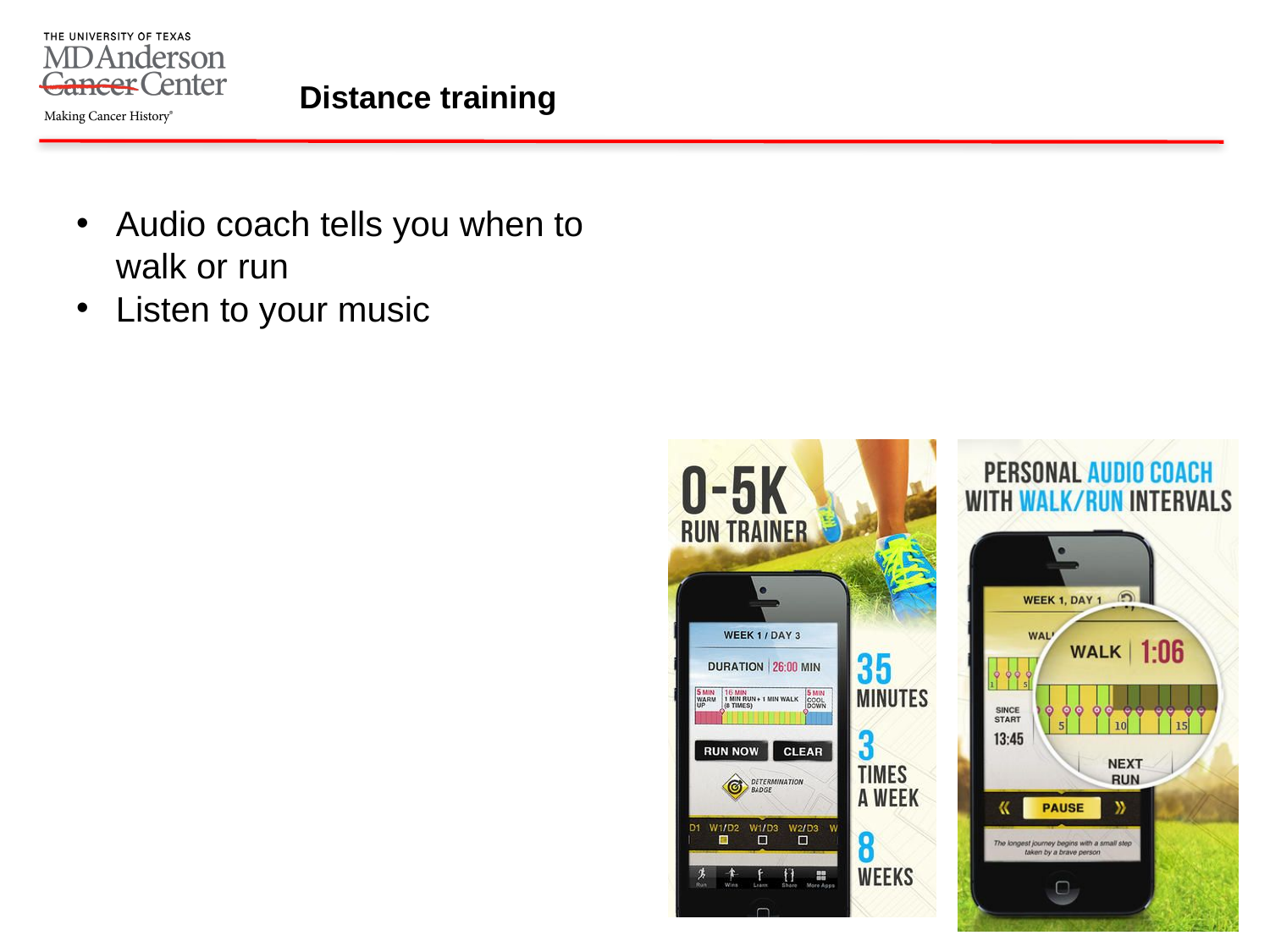

Distance training
Audio coach tells you when to walk or run
Listen to your music

## Slide 5
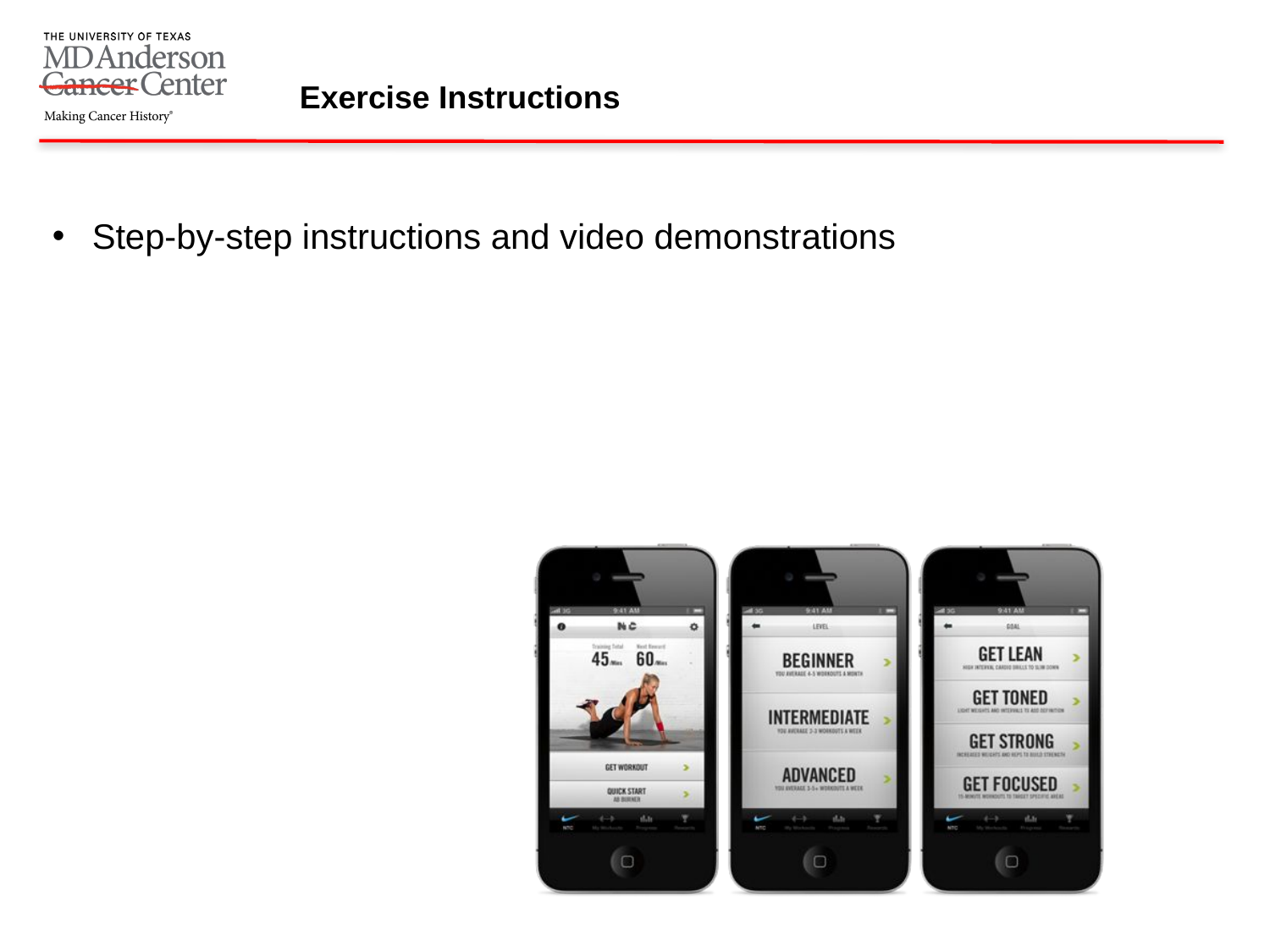

Exercise Instructions
Step-by-step instructions and video demonstrations

## Slide 6
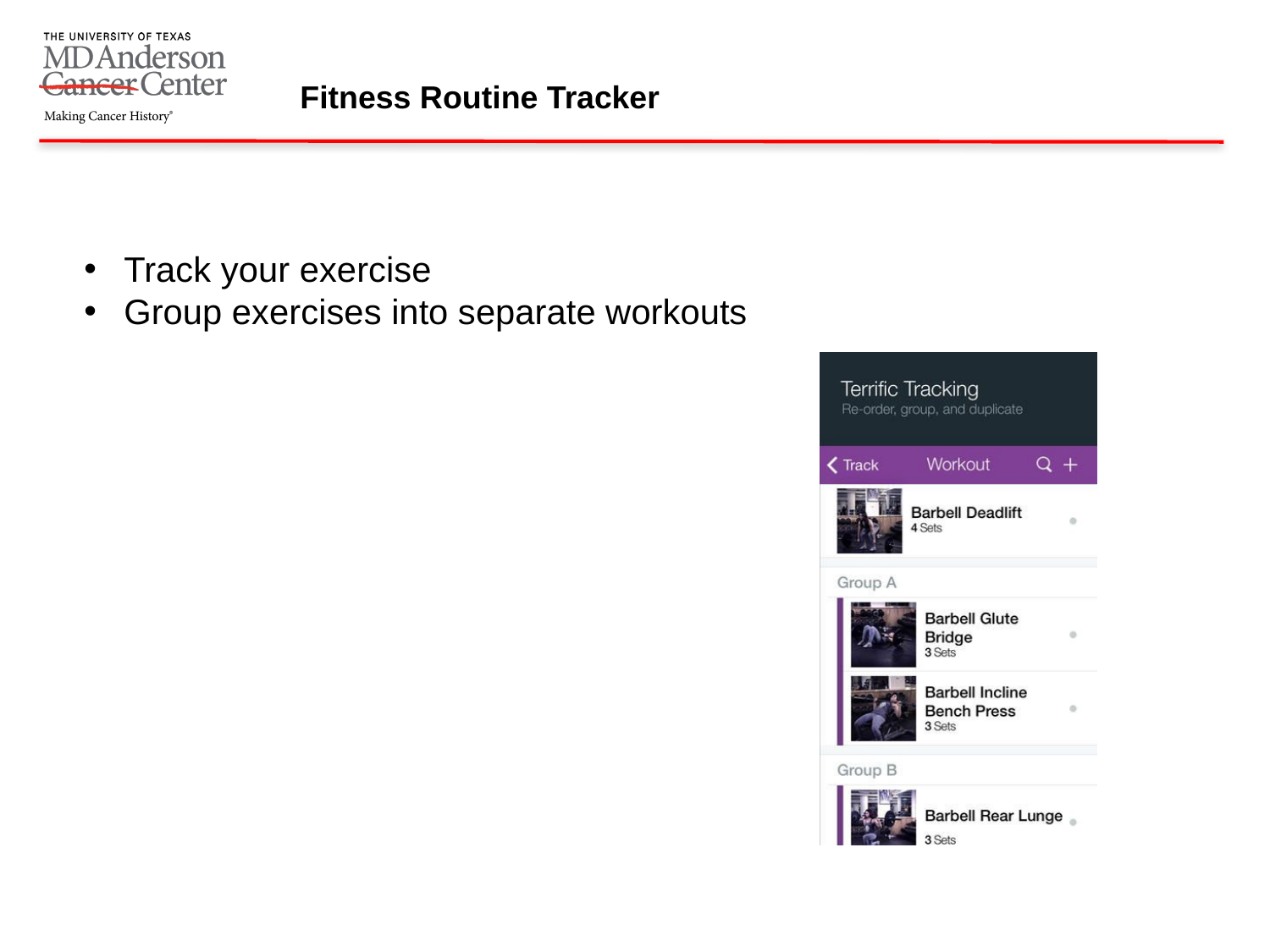

Fitness Routine Tracker
Track your exercise
Group exercises into separate workouts

## Slide 7
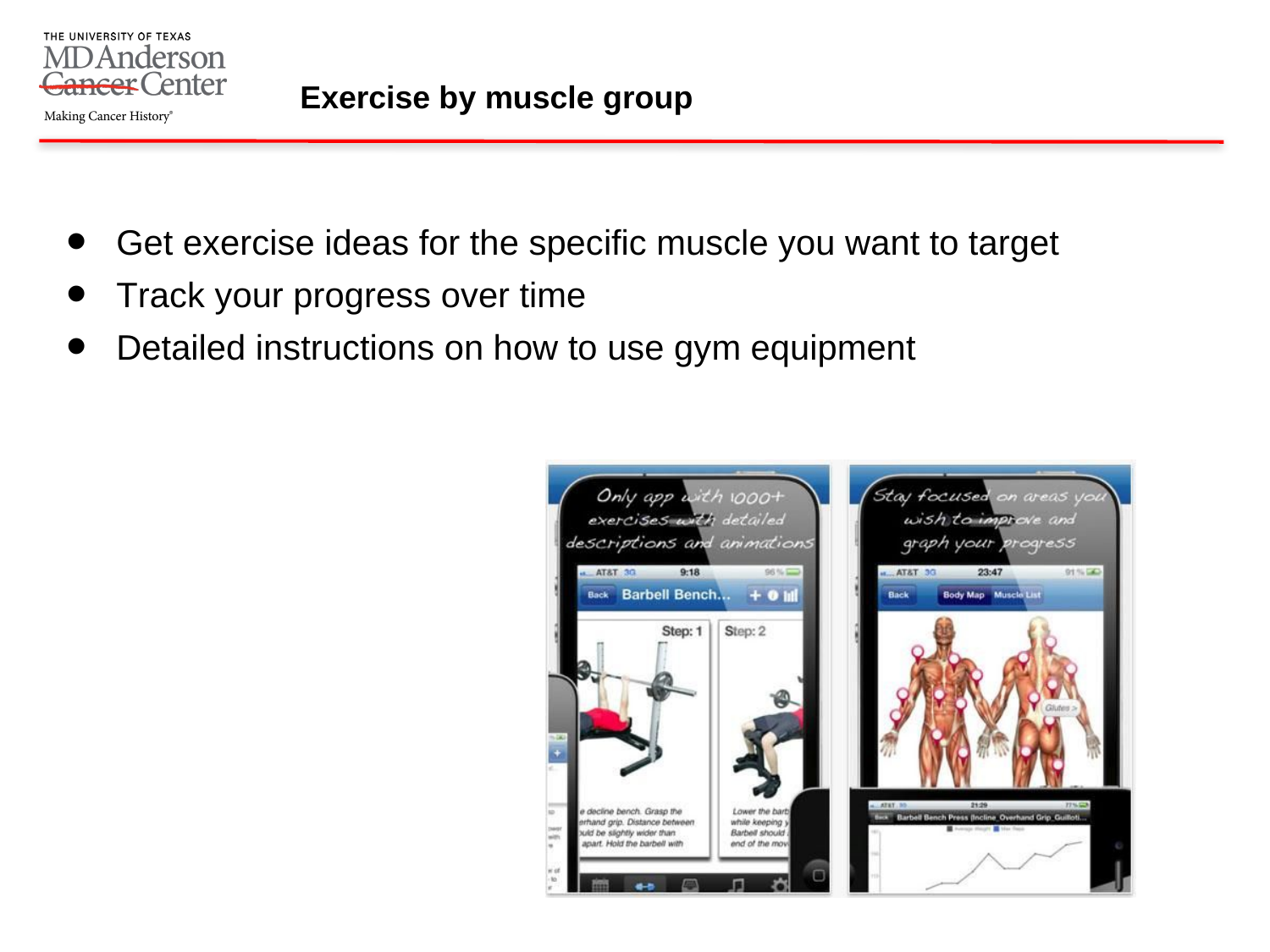

Exercise by muscle group
Get exercise ideas for the specific muscle you want to target
Track your progress over time
Detailed instructions on how to use gym equipment

## Slide 8
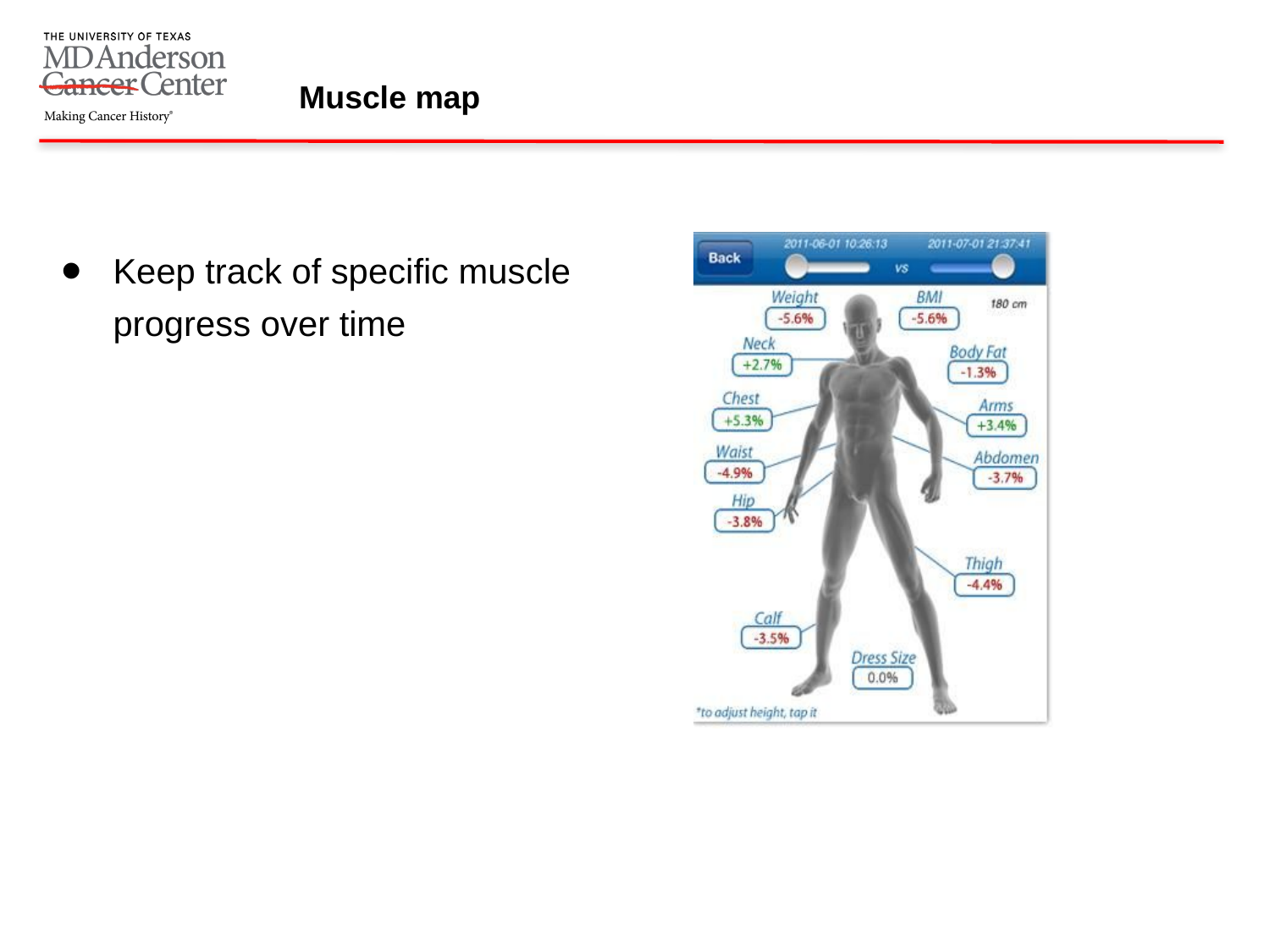

Muscle map
Keep track of specific muscle progress over time

## Slide 9
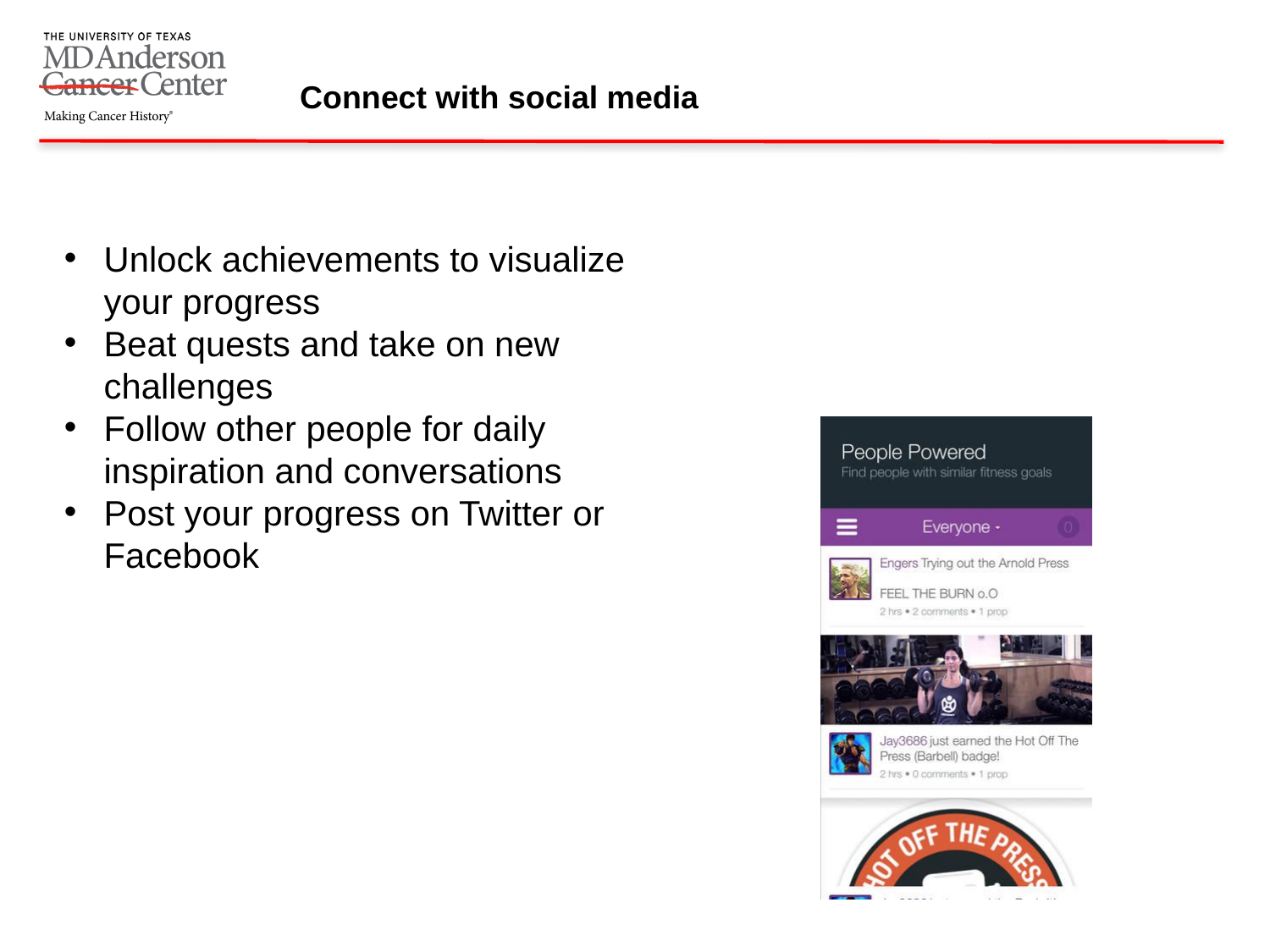

Connect with social media
Unlock achievements to visualize your progress
Beat quests and take on new challenges
Follow other people for daily inspiration and conversations
Post your progress on Twitter or Facebook

## Slide 10
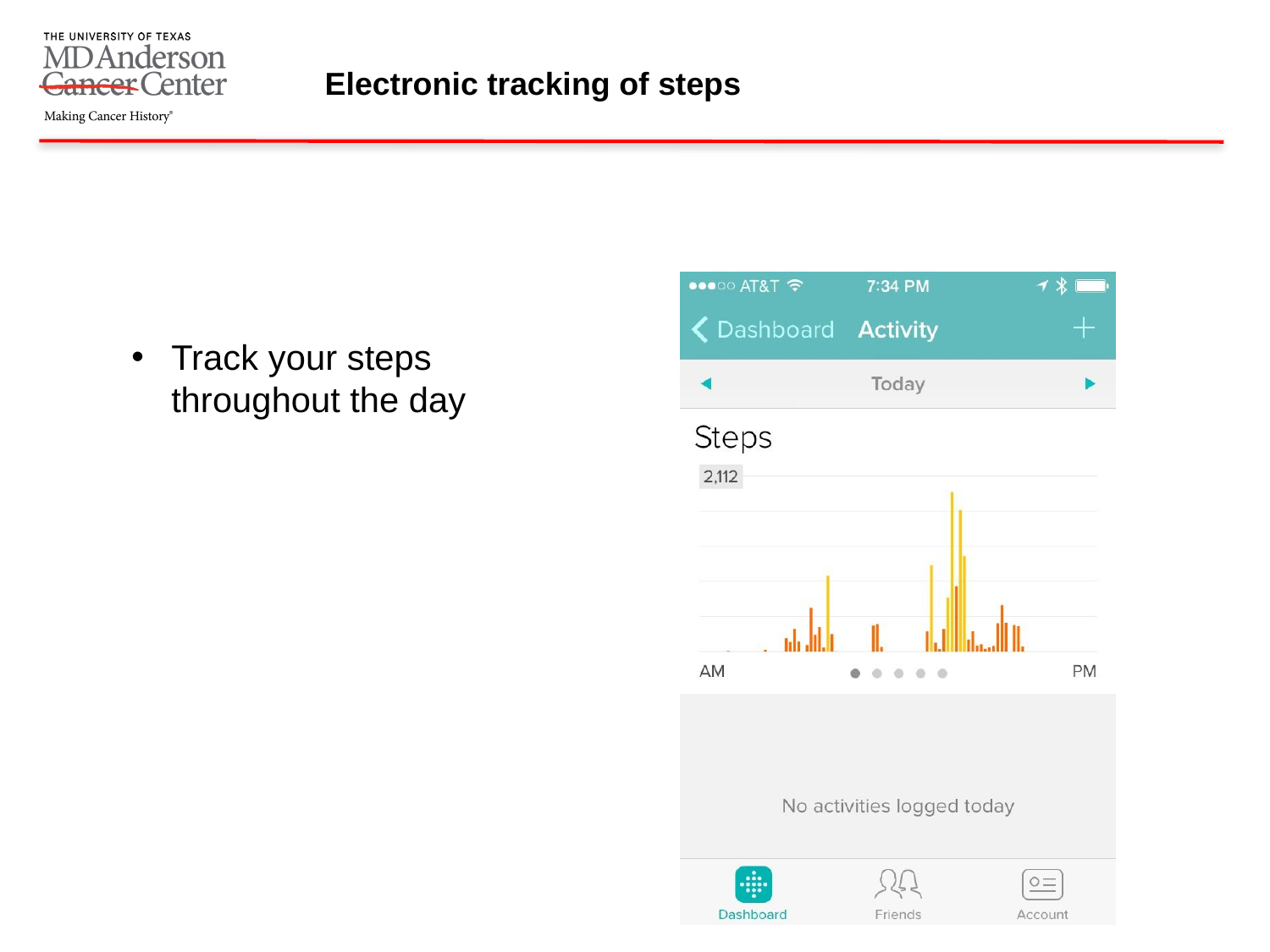

Electronic tracking of steps
Track your steps throughout the day

## Slide 11
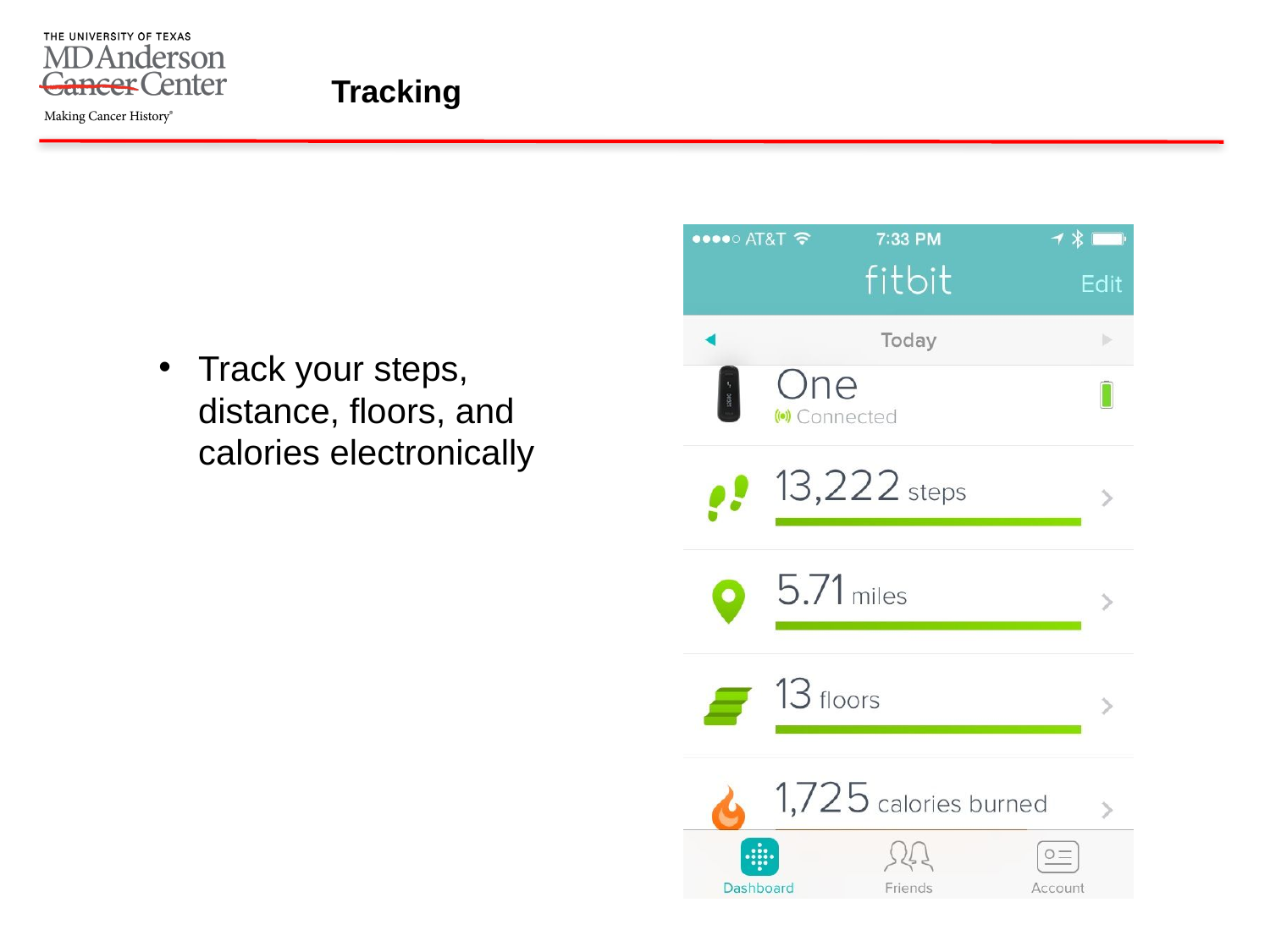

Tracking
Track your steps, distance, floors, and calories electronically

## Slide 12
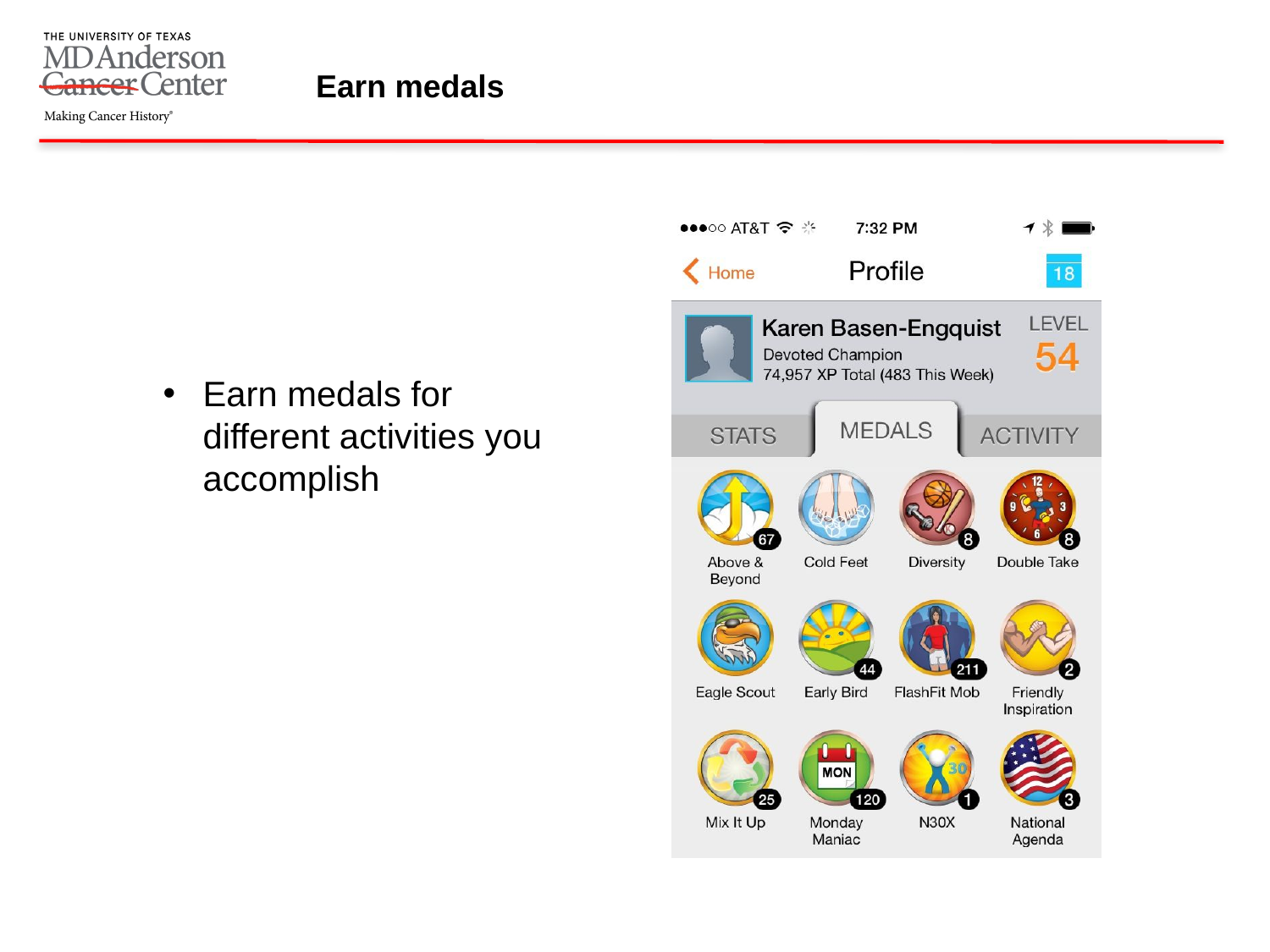

Earn medals
Earn medals for different activities you accomplish

## Slide 13
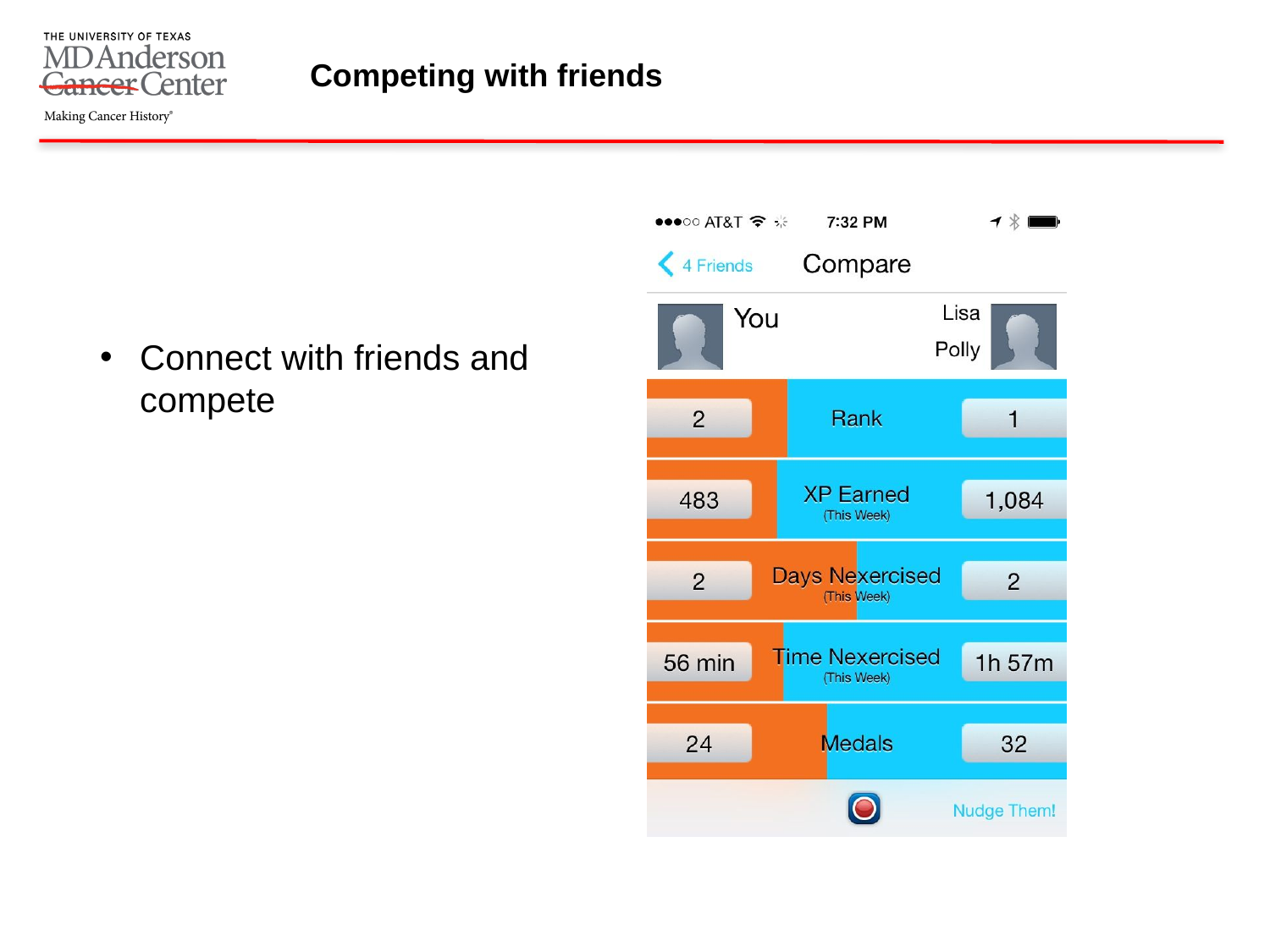

Competing with friends
Connect with friends and compete

## Slide 14
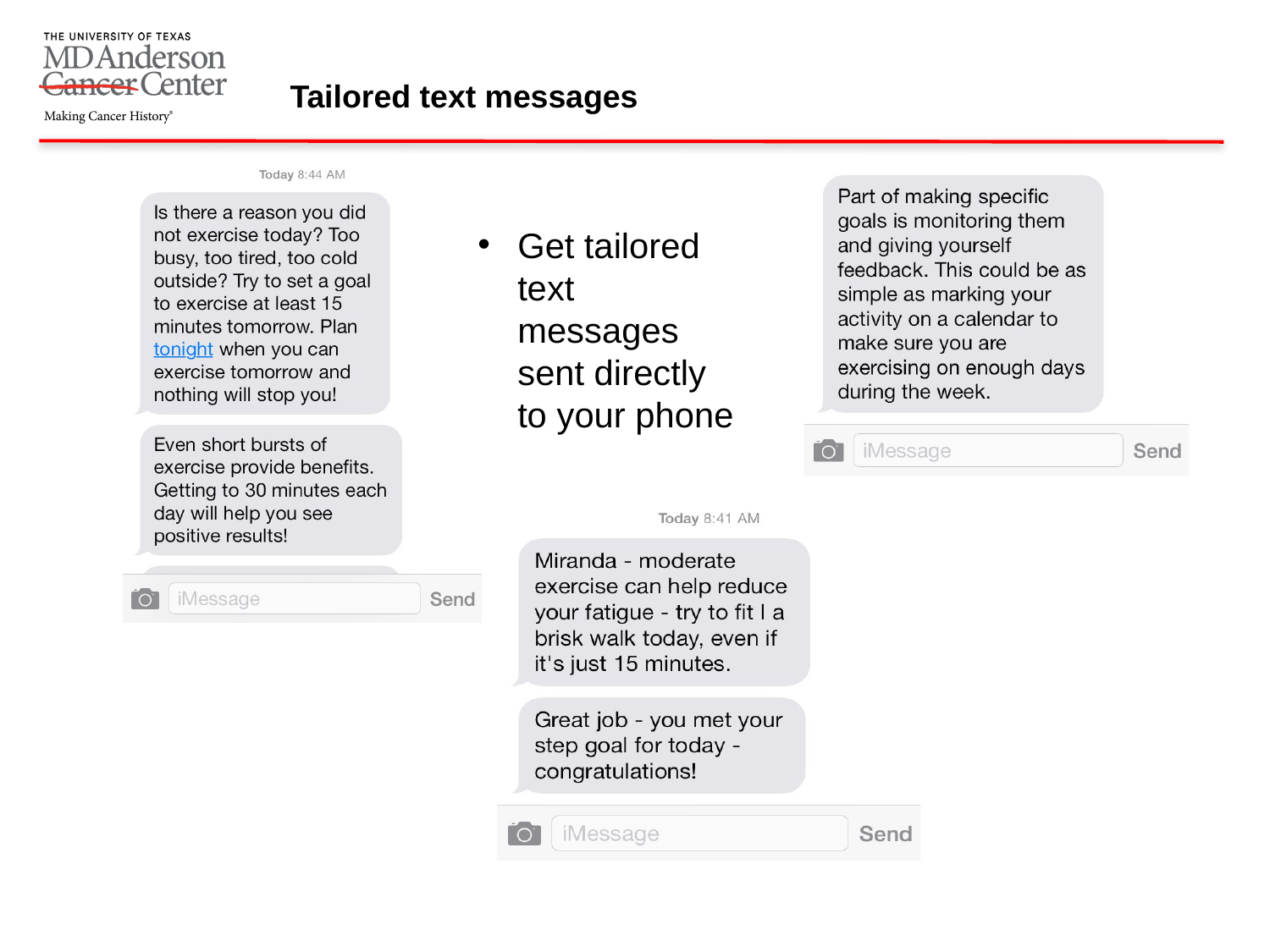

Tailored text messages
Get tailored text messages sent directly to your phone
