# Supplementary material for: Mobile Health Physical Activity Intervention Preferences in Cancer Survivors: A Qualitative Study
Source: JMIR Mhealth Uhealth. 2017 Jan 24;5(1):e3. doi: 10.2196/mhealth.6970 (PMC5296620; doi:10.2196/mhealth.6970)
Supplement: Multimedia Appendix 3 [file mhealth_v5i1e3_app3.pdf]

Ranking	App Feature	N Comments	Percentage Positive	Description of App Feature
1	Exercise by Muscle Group	8	100%	Get exercise ideas for the specific muscle you want to target, track your progress over time, receive detailed instructions on how to use gym equipment
2	Electronic Tracking of Steps	33	97%	Track your steps throughout the day, track your steps, distance, floors, and calories electronically
3	Tailored Text Messages	28	93%	Get tailored text messages sent directly to your phone
4	Exercise Instruction	15	87%	Step-by-step instructions and video demonstrations
5	GPS Tracker	14	71%	View the route of your run/walk, track distance, pace, and duration
6	Measurement Tracker	14	71%	Track your measurements over time, track hip, waist, and neck circumference
7	Fitness Routine Tracker	13	69%	Track your exercise, group exercises into separate workouts
8	Earn Medals	17	65%	Earn medals for different activities you accomplish
9	Distance Training	16	63%	Audio coach tells you when to walk or run, listen to your music
10	Competing with Friends	14	57%	Connect with friends and compete
11	Muscle Map	10	50%	Keep track of specific muscle progress over time
12	Connect with Social Media	20	25%	Post your progress on Twitter or Facebook
