# Supplementary material for: Mobile Health Physical Activity Intervention Preferences in Cancer Survivors: A Qualitative Study
Source: JMIR Mhealth Uhealth. 2017 Jan 24;5(1):e3. doi: 10.2196/mhealth.6970 (PMC5296620; doi:10.2196/mhealth.6970)
Supplement: Multimedia Appendix 4 [file mhealth_v5i1e3_app4.pptx]

## Slide 1
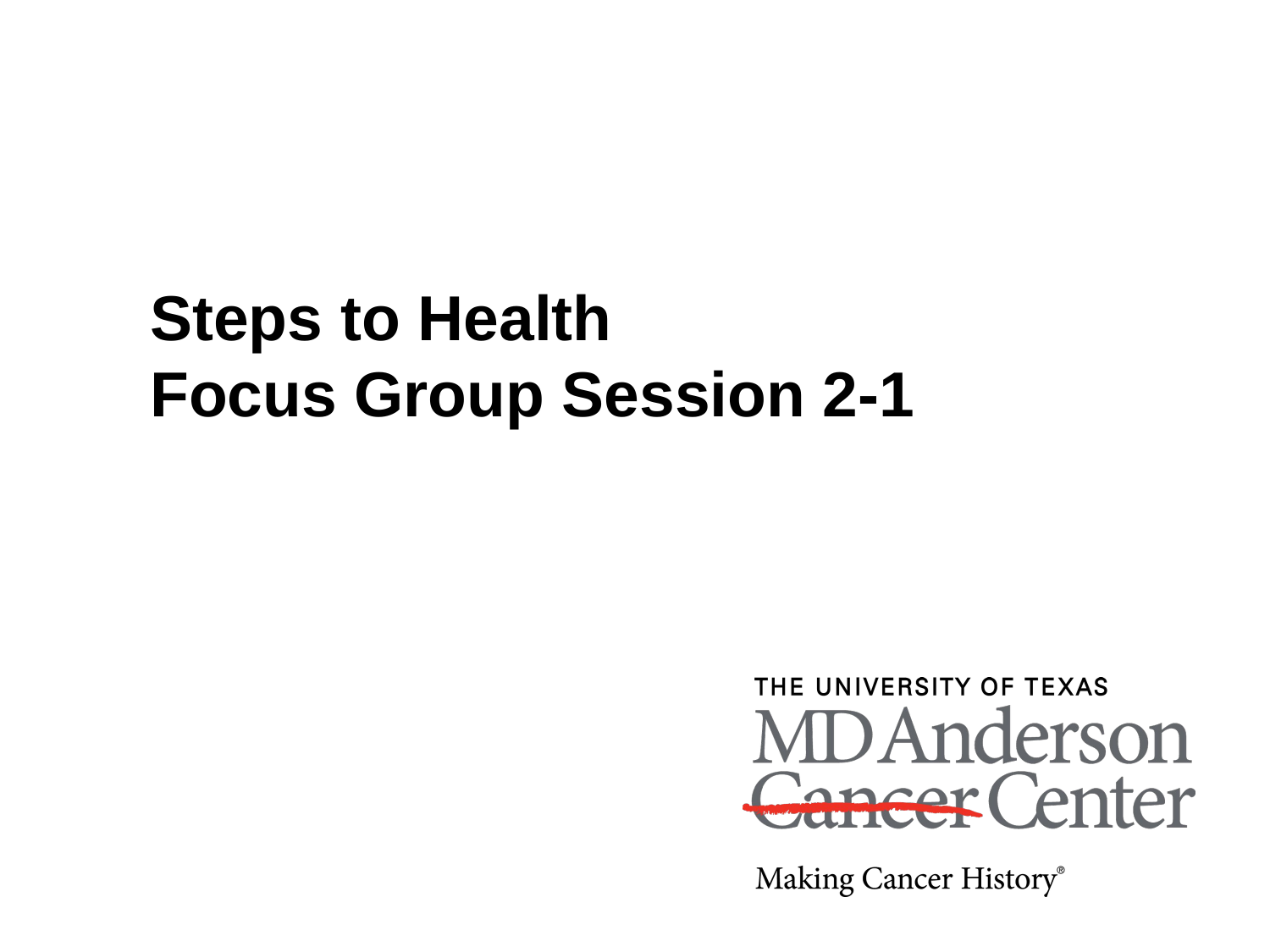

Steps to Health
Focus Group Session 2-1

## Slide 2
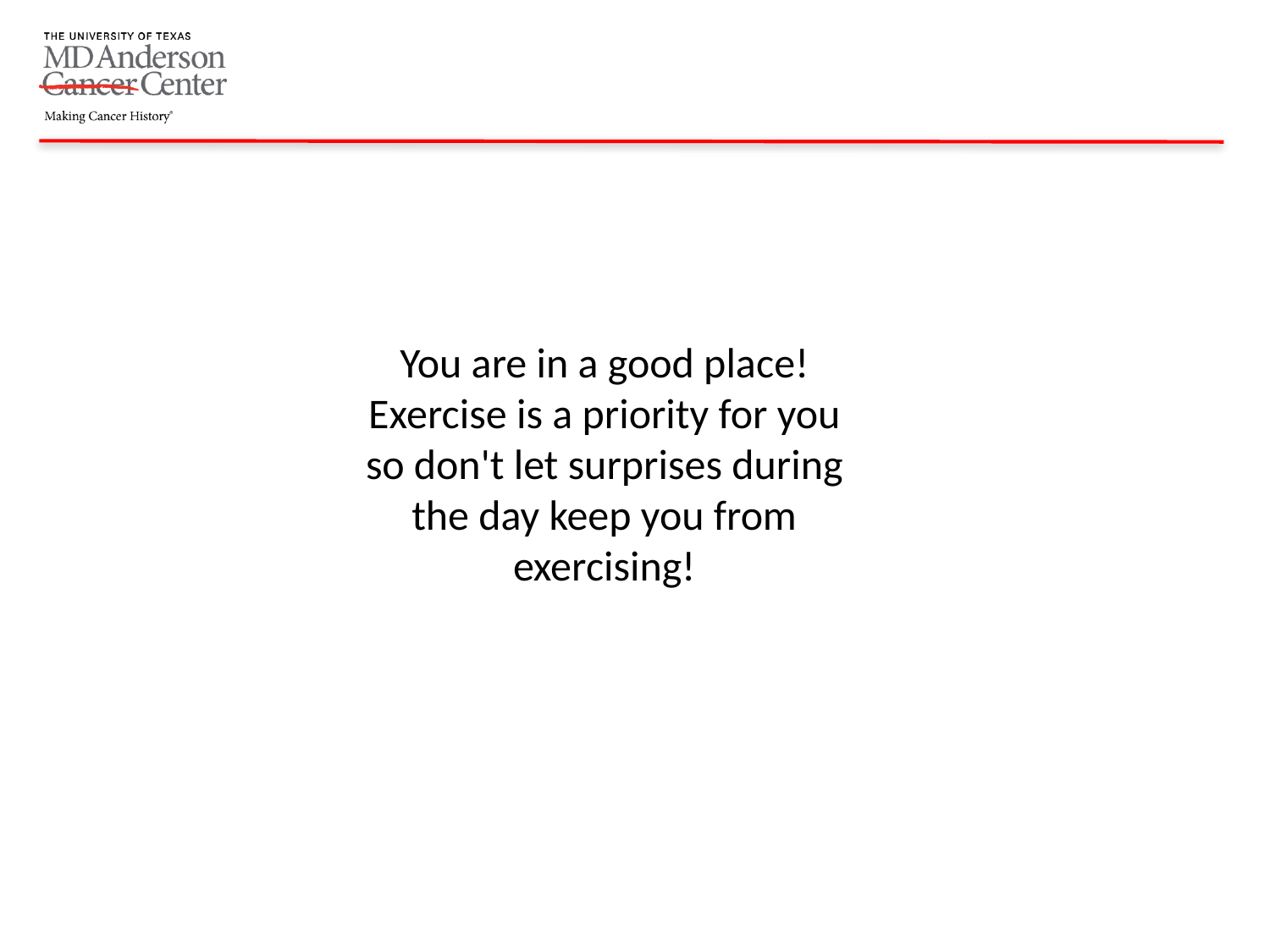

You are in a good place! Exercise is a priority for you so don't let surprises during the day keep you from exercising!

## Slide 3
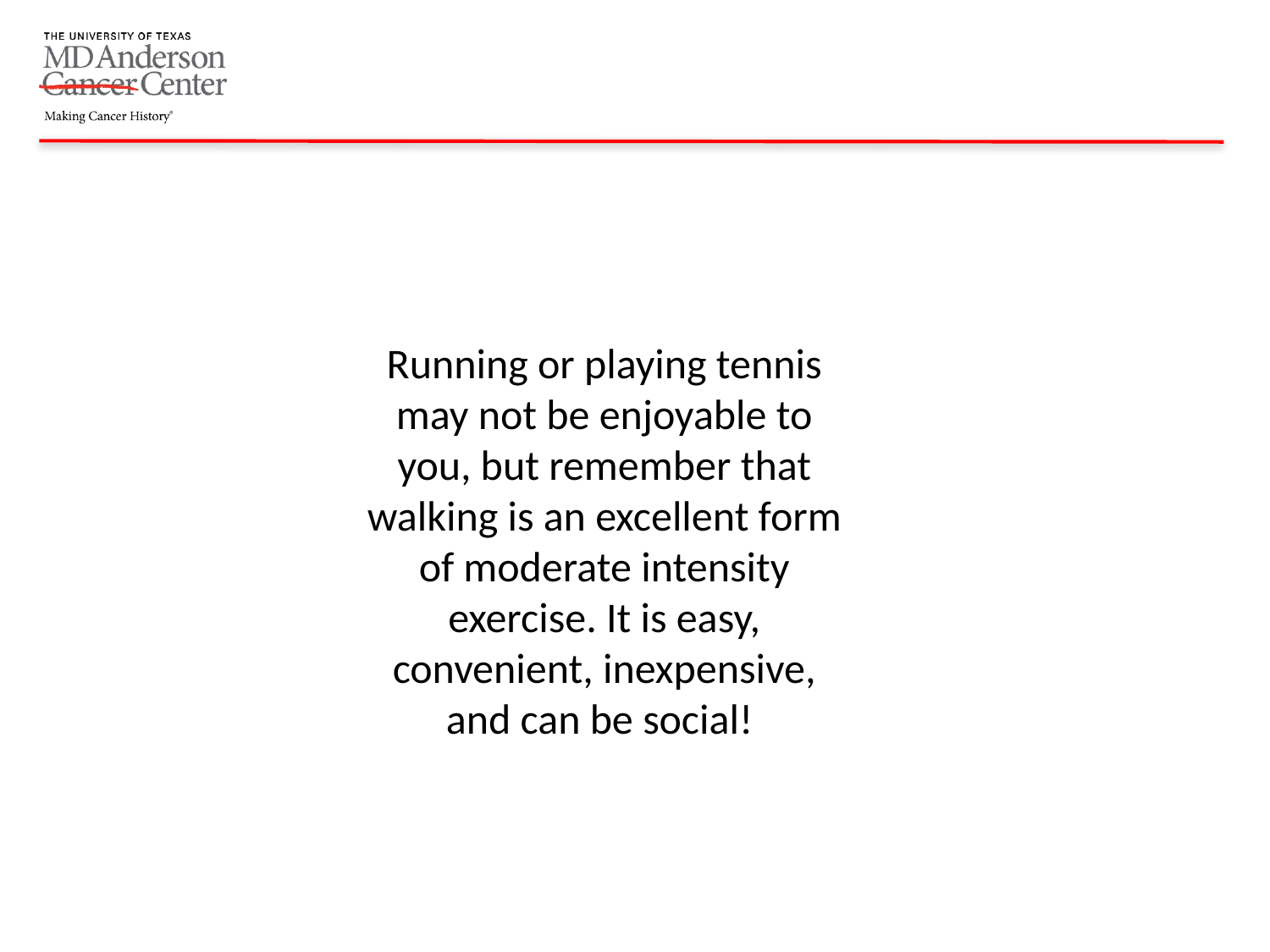

Running or playing tennis may not be enjoyable to you, but remember that walking is an excellent form of moderate intensity exercise. It is easy, convenient, inexpensive, and can be social!

## Slide 4
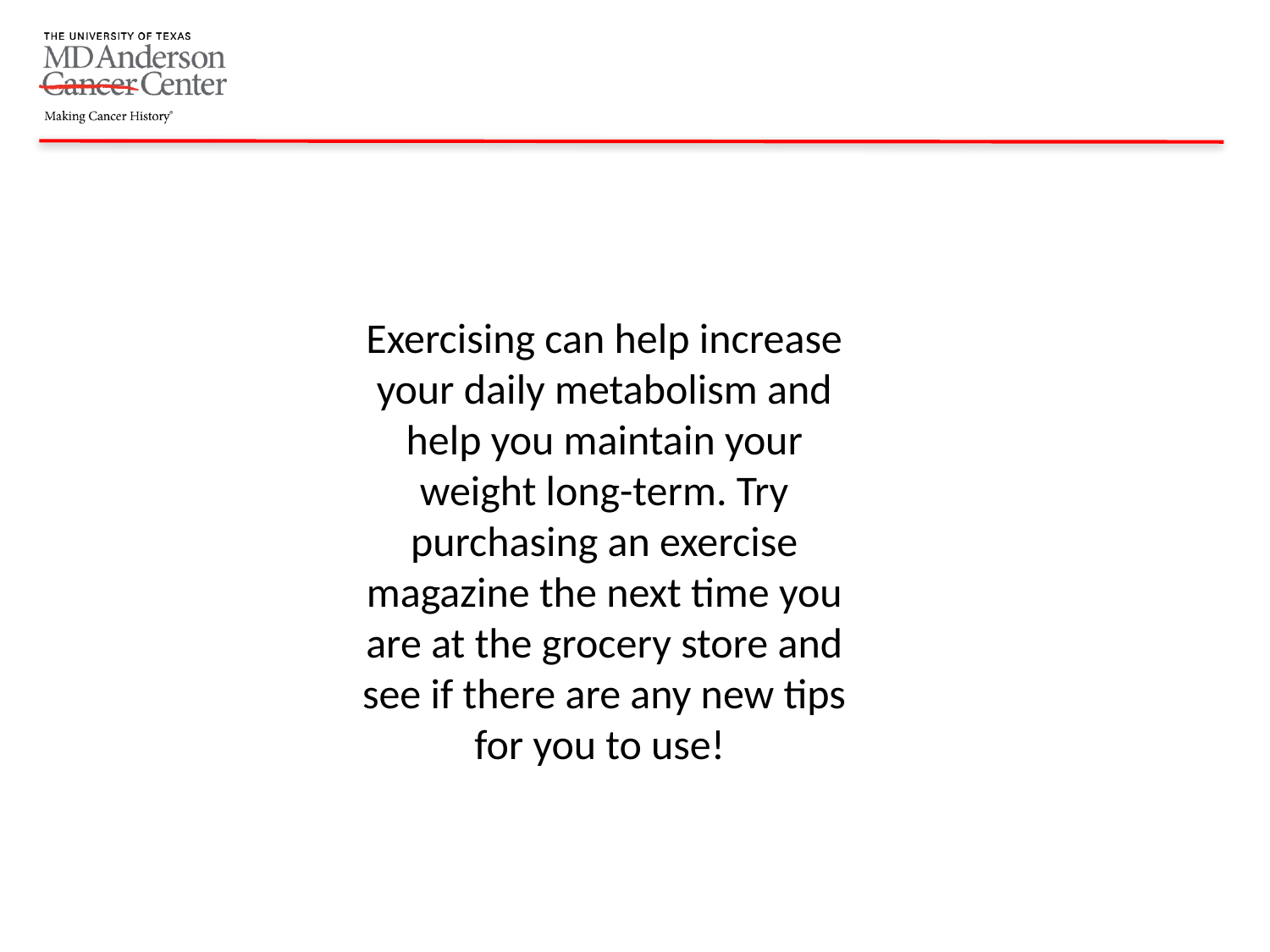

Exercising can help increase your daily metabolism and help you maintain your weight long-term. Try purchasing an exercise magazine the next time you are at the grocery store and see if there are any new tips for you to use!

## Slide 5
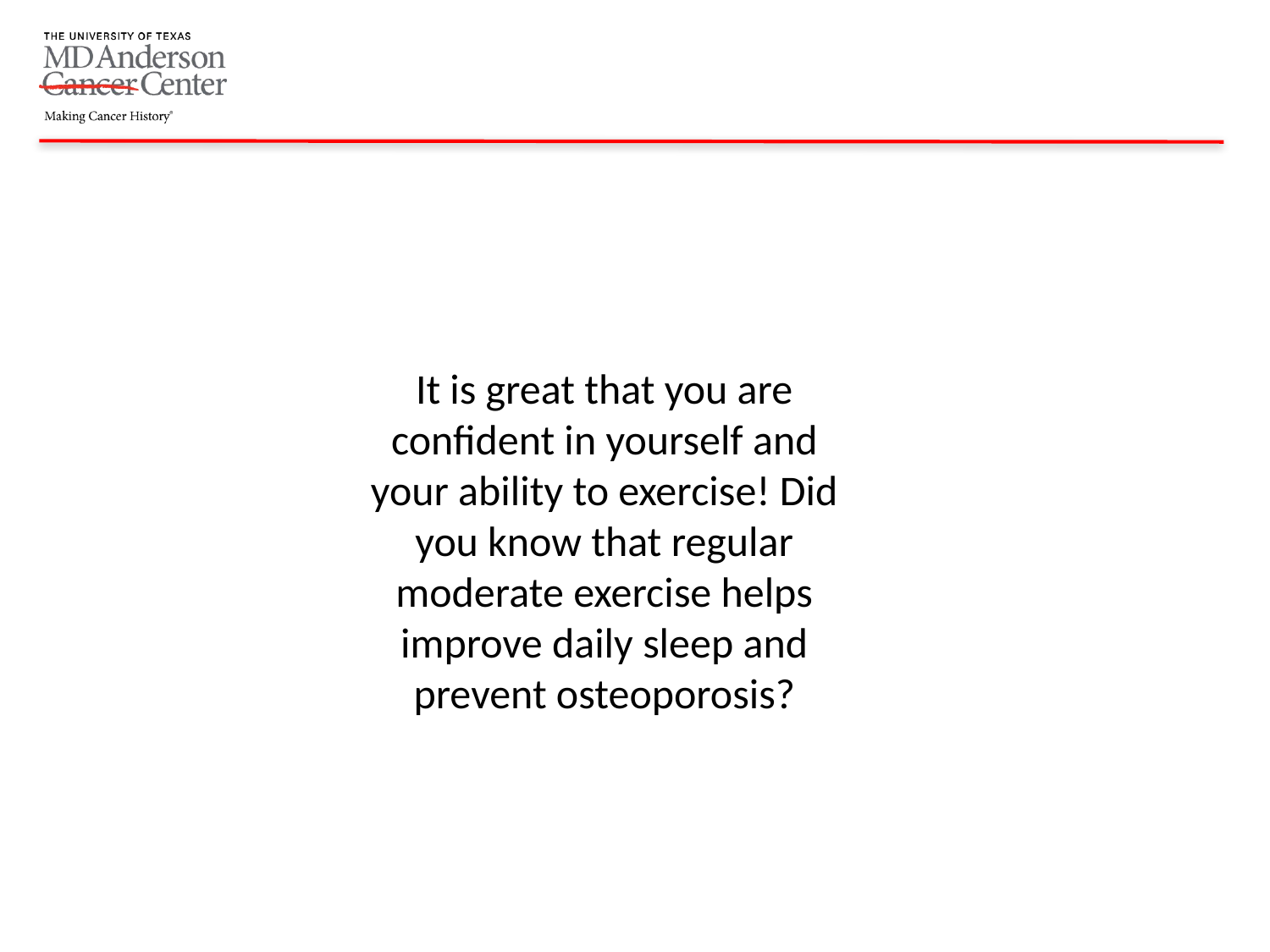

It is great that you are confident in yourself and your ability to exercise! Did you know that regular moderate exercise helps improve daily sleep and prevent osteoporosis?

## Slide 6
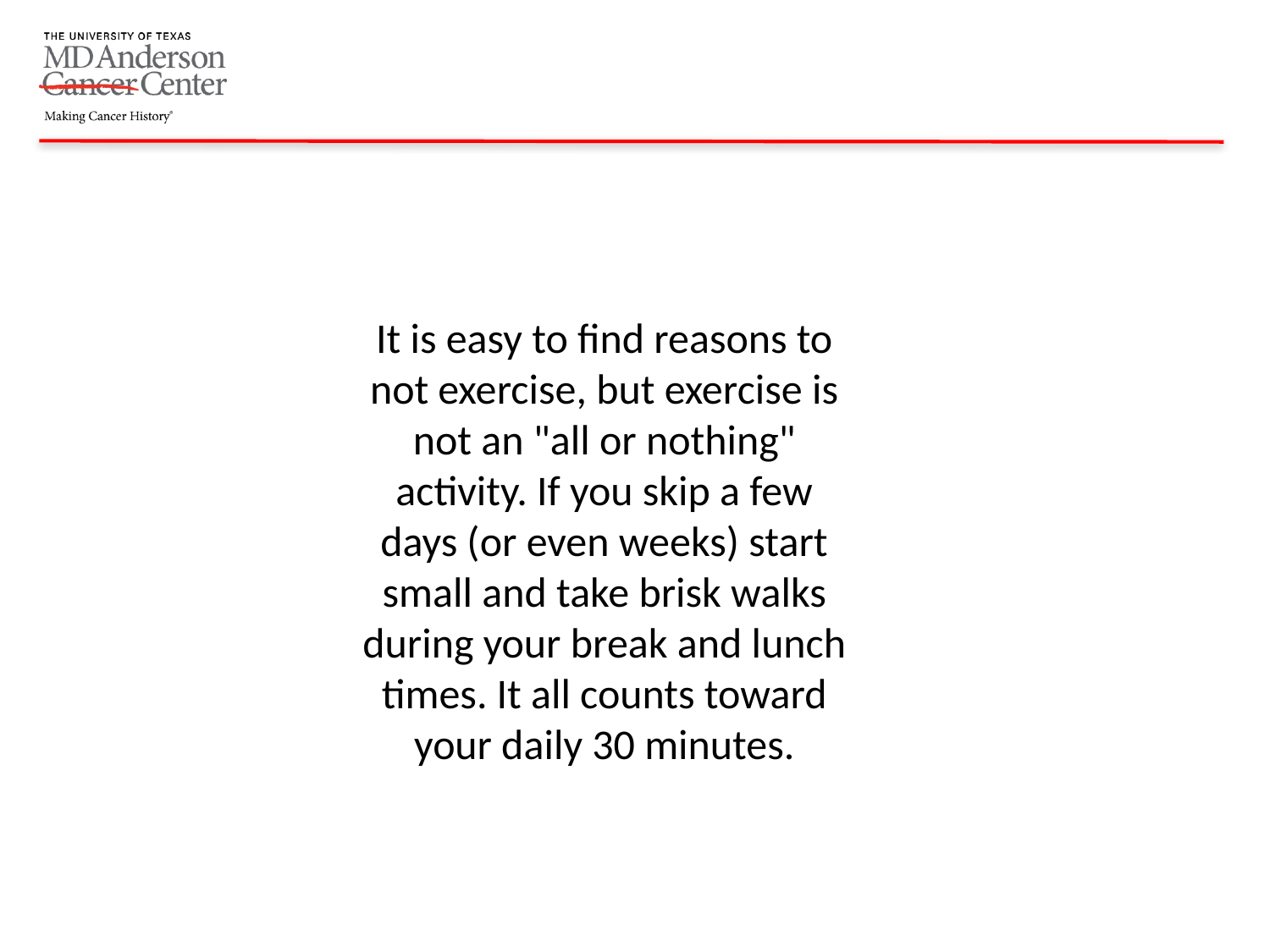

It is easy to find reasons to not exercise, but exercise is not an "all or nothing" activity. If you skip a few days (or even weeks) start small and take brisk walks during your break and lunch times. It all counts toward your daily 30 minutes.

## Slide 7
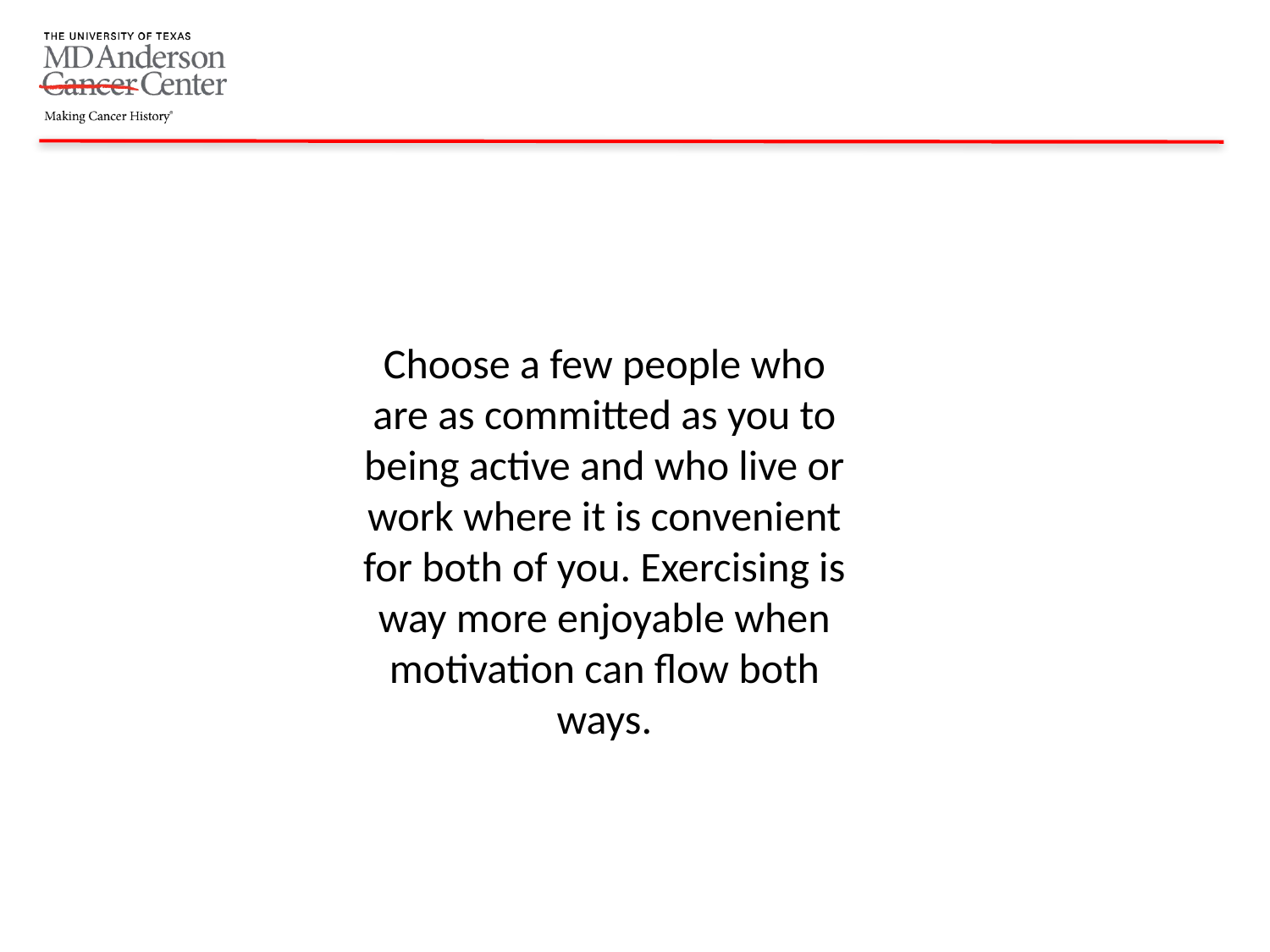

Choose a few people who are as committed as you to being active and who live or work where it is convenient for both of you. Exercising is way more enjoyable when motivation can flow both ways.

## Slide 8
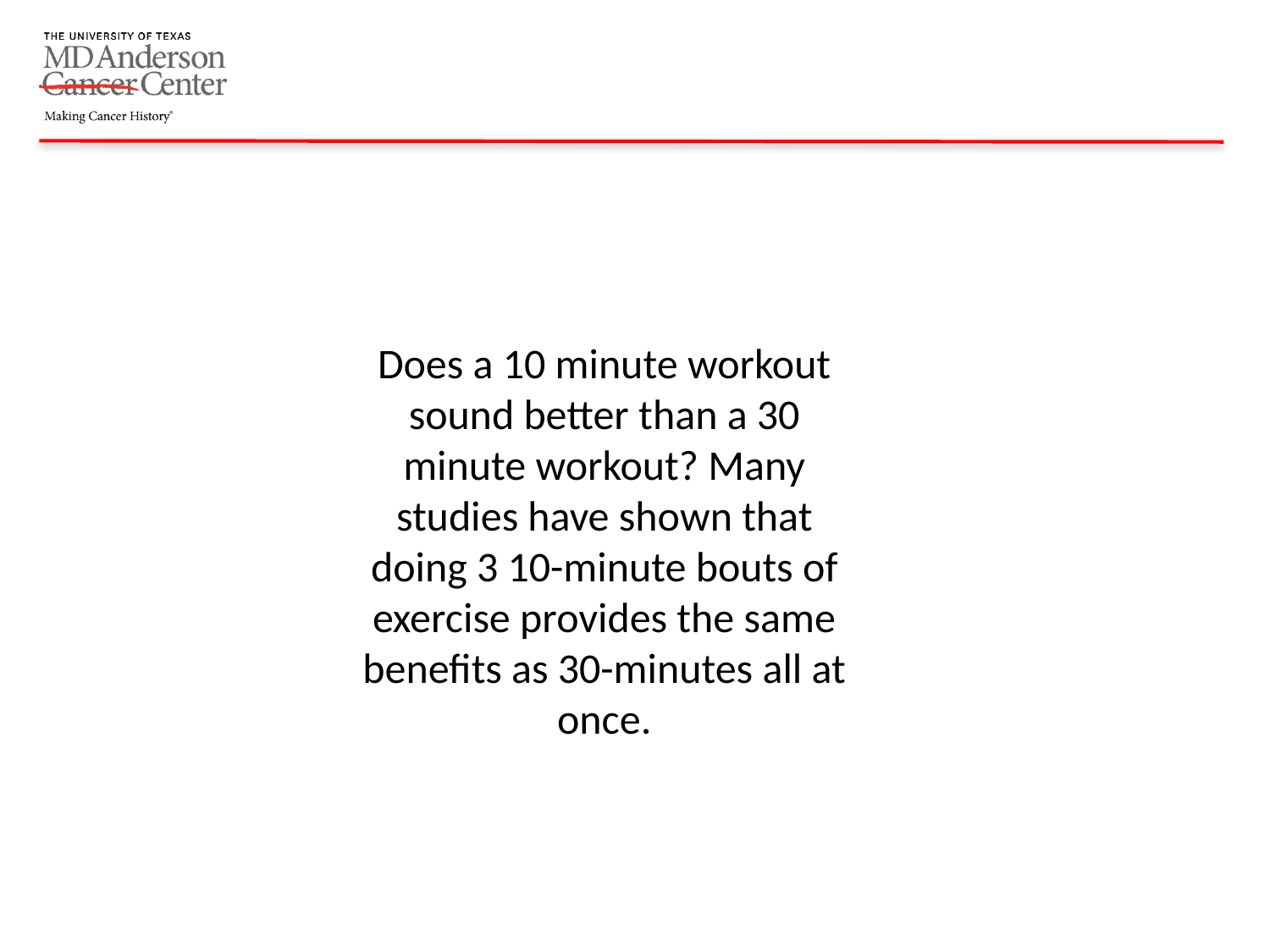

Does a 10 minute workout sound better than a 30 minute workout? Many studies have shown that doing 3 10-minute bouts of exercise provides the same benefits as 30-minutes all at once.

## Slide 9
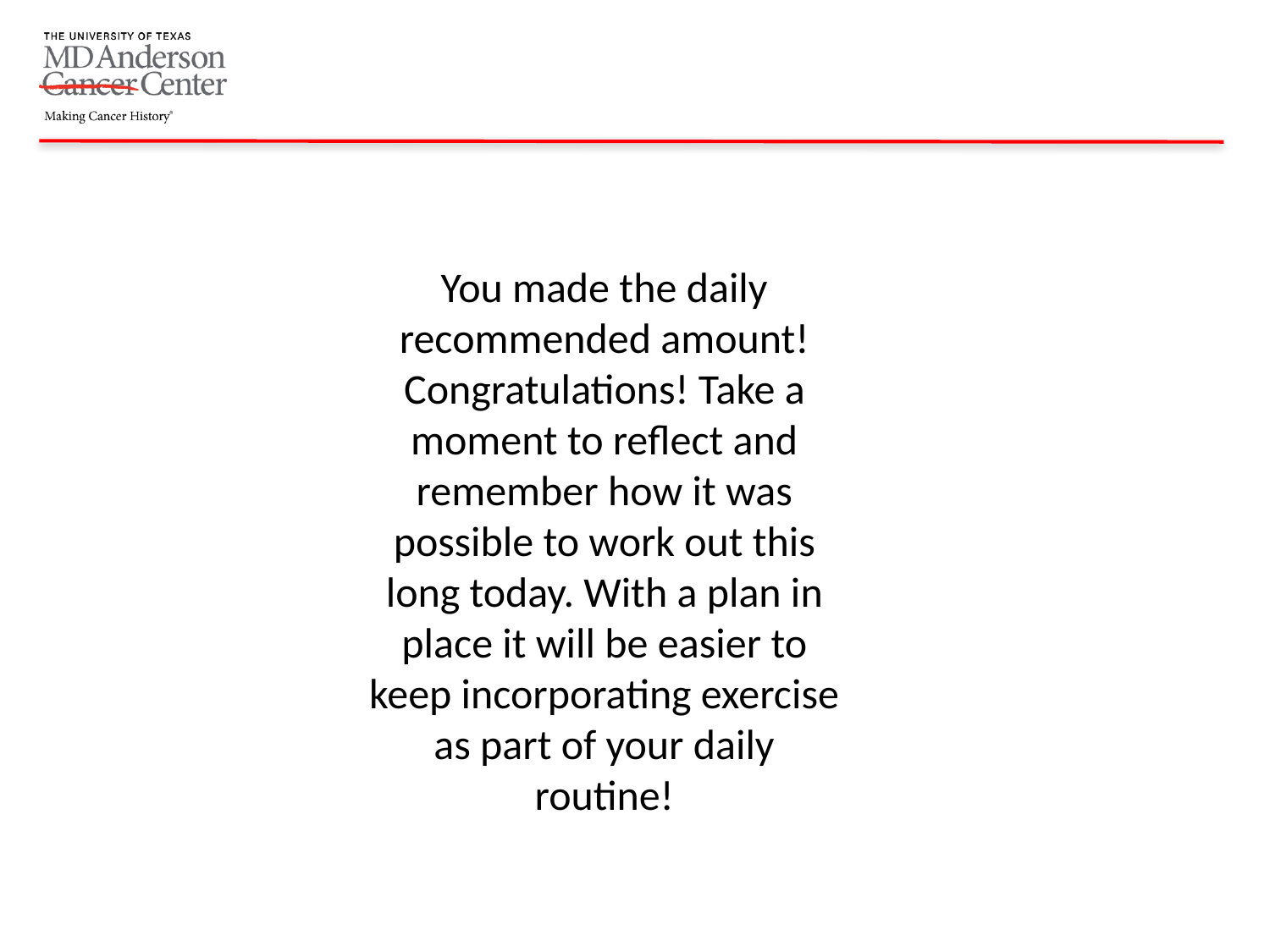

You made the daily recommended amount! Congratulations! Take a moment to reflect and remember how it was possible to work out this long today. With a plan in place it will be easier to keep incorporating exercise as part of your daily routine!

## Slide 10
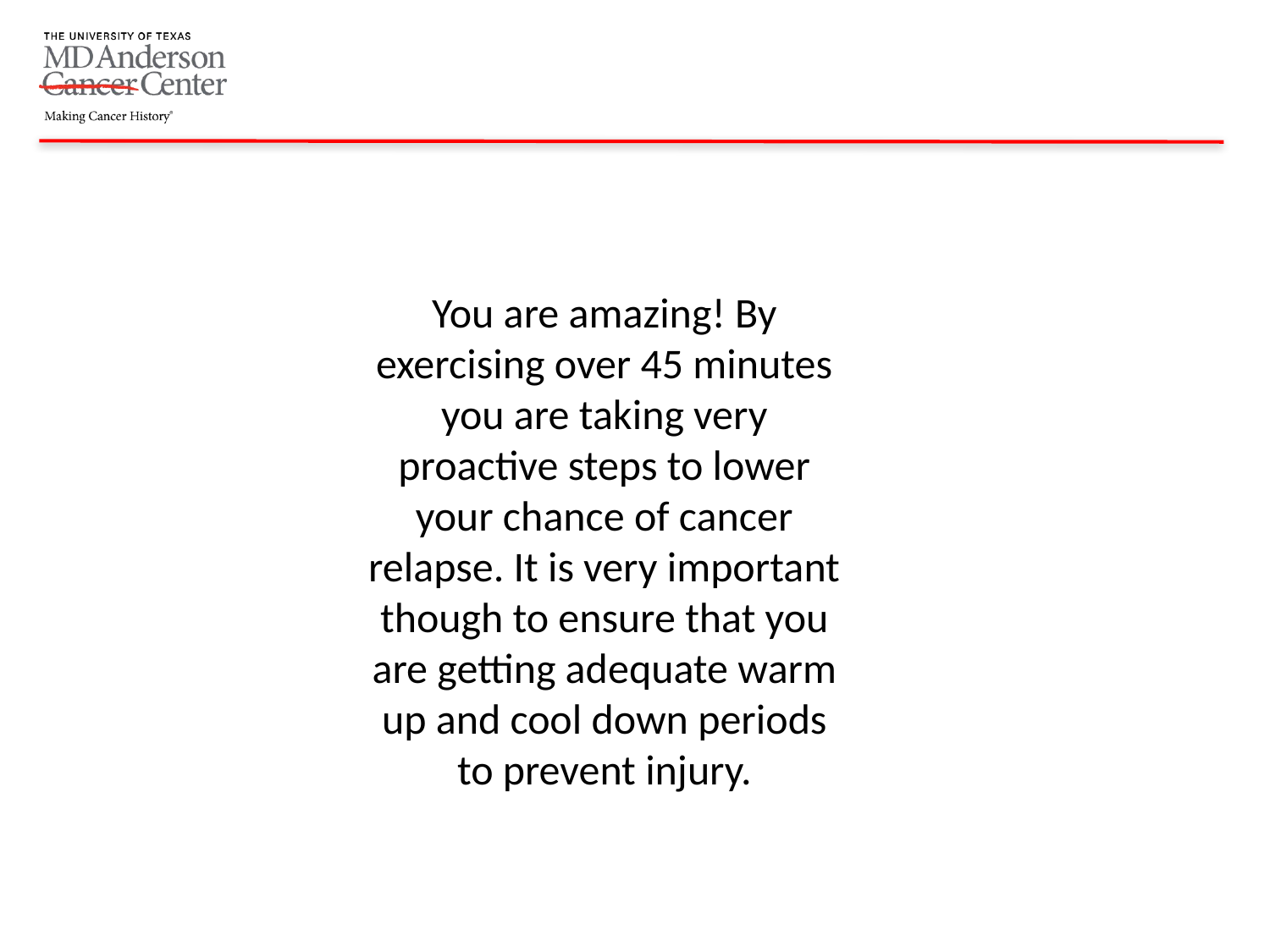

You are amazing! By exercising over 45 minutes you are taking very proactive steps to lower your chance of cancer relapse. It is very important though to ensure that you are getting adequate warm up and cool down periods to prevent injury.

## Slide 11
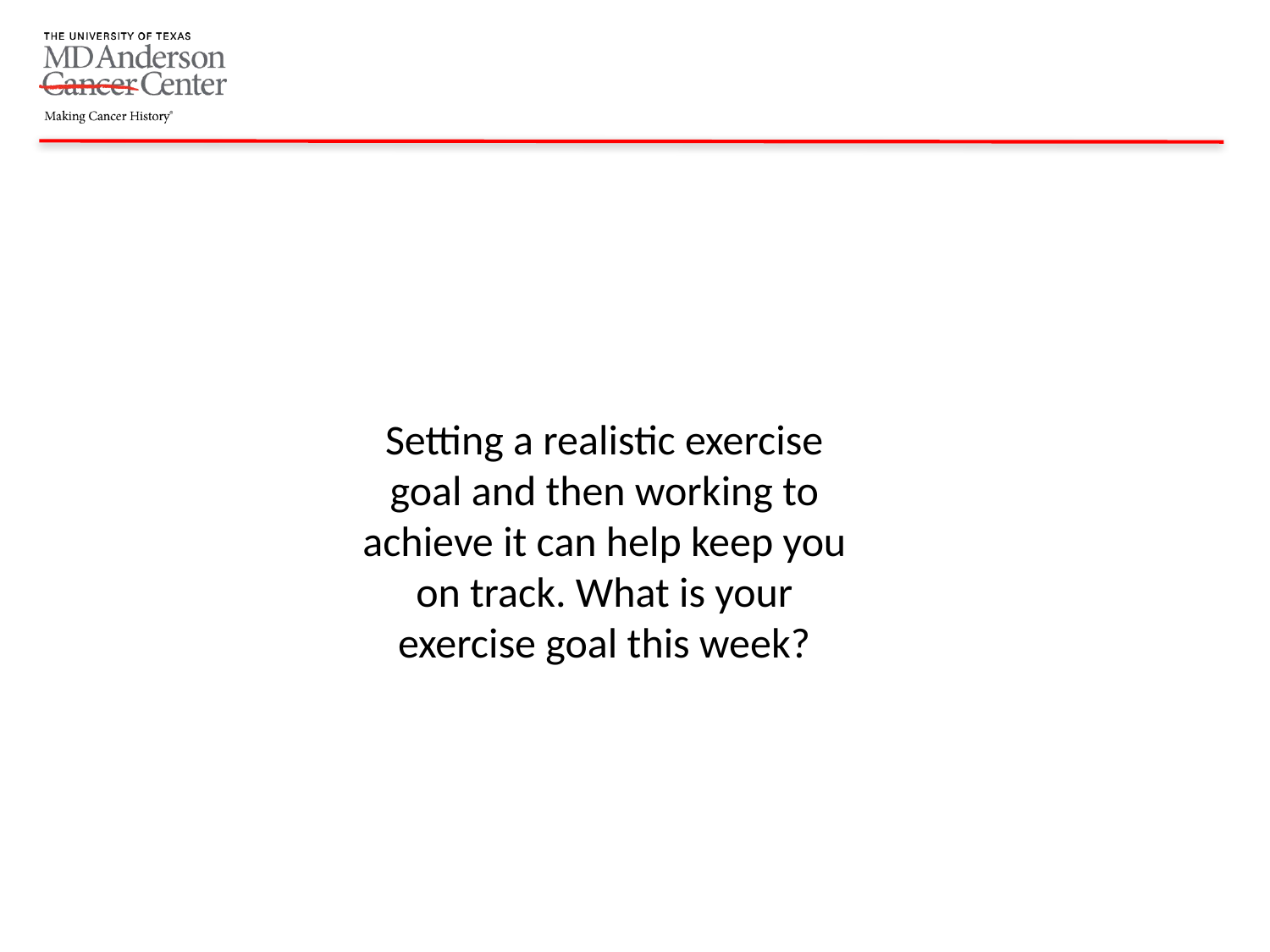

Setting a realistic exercise goal and then working to achieve it can help keep you on track. What is your exercise goal this week?

## Slide 12
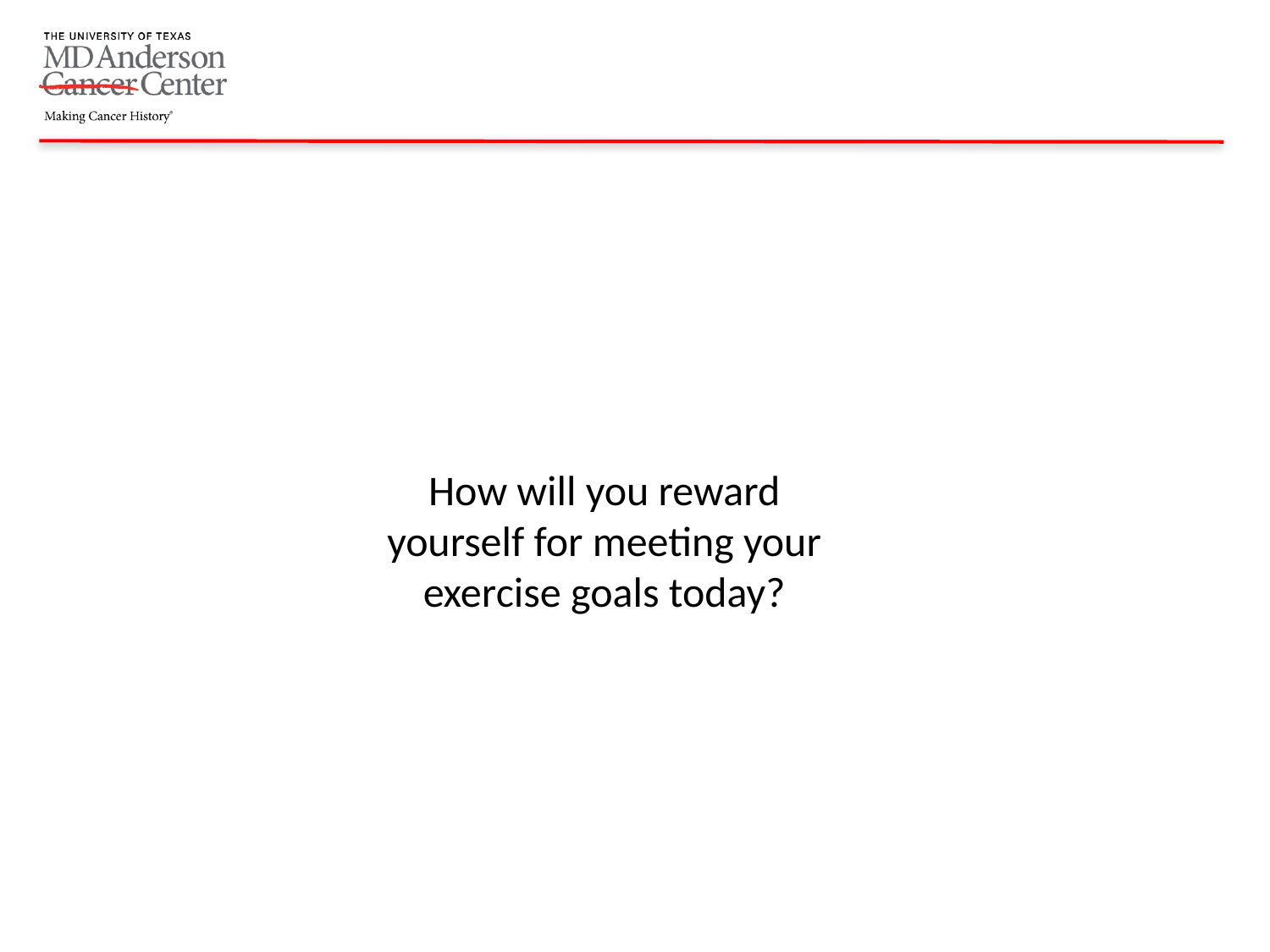

How will you reward yourself for meeting your exercise goals today?

## Slide 13
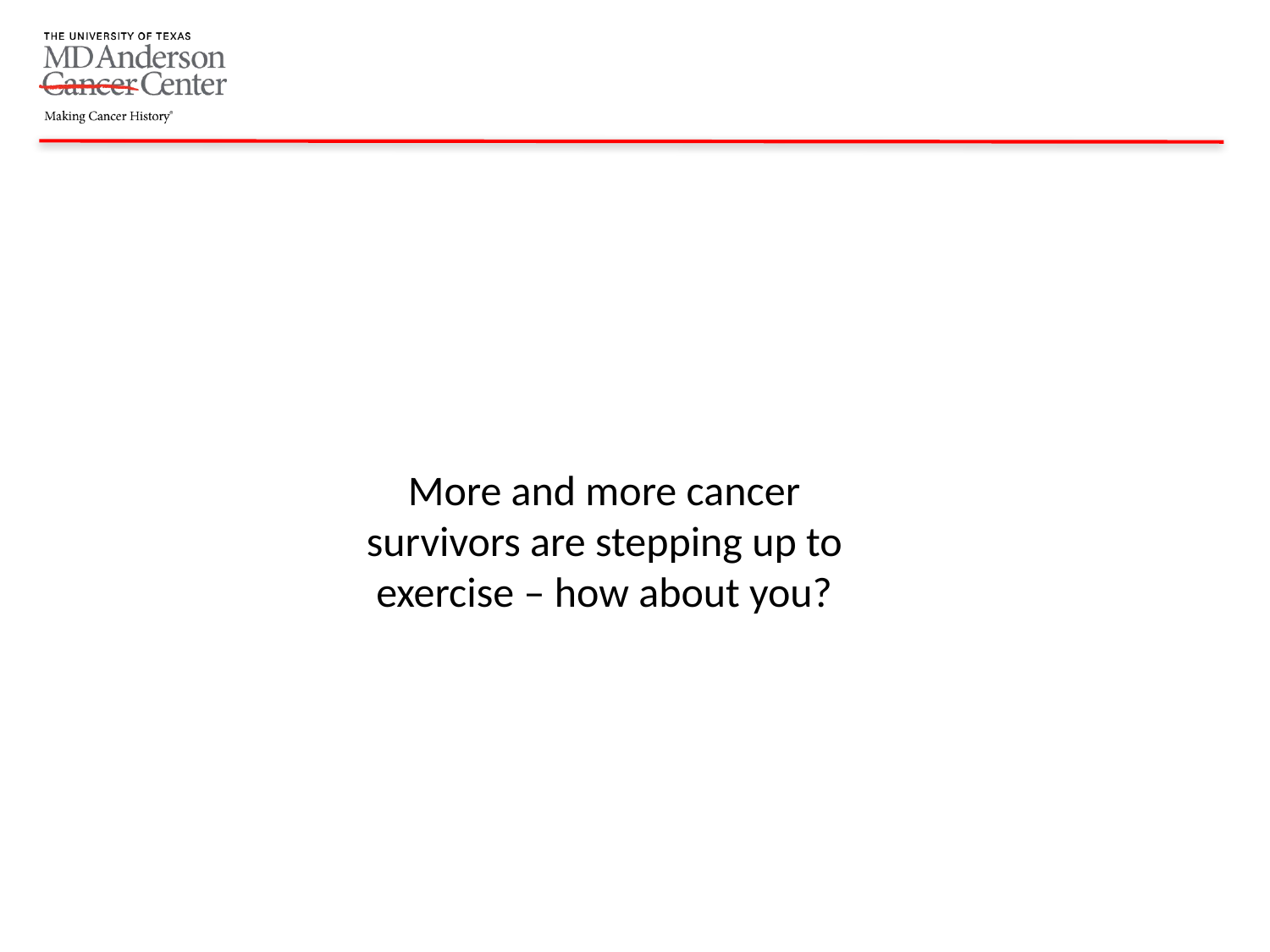

More and more cancer survivors are stepping up to exercise – how about you?

## Slide 14
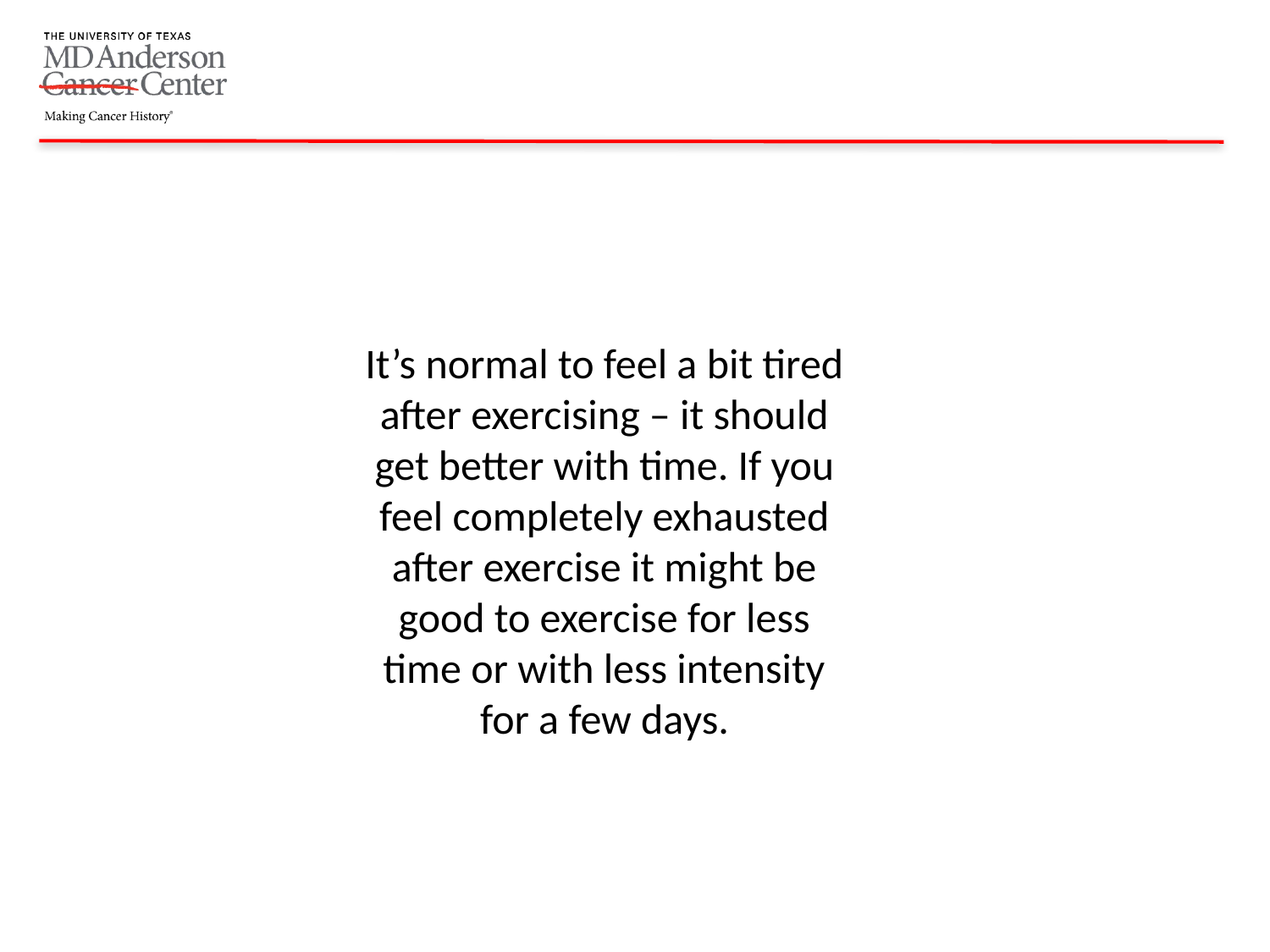

It’s normal to feel a bit tired after exercising – it should get better with time. If you feel completely exhausted after exercise it might be good to exercise for less time or with less intensity for a few days.

## Slide 15
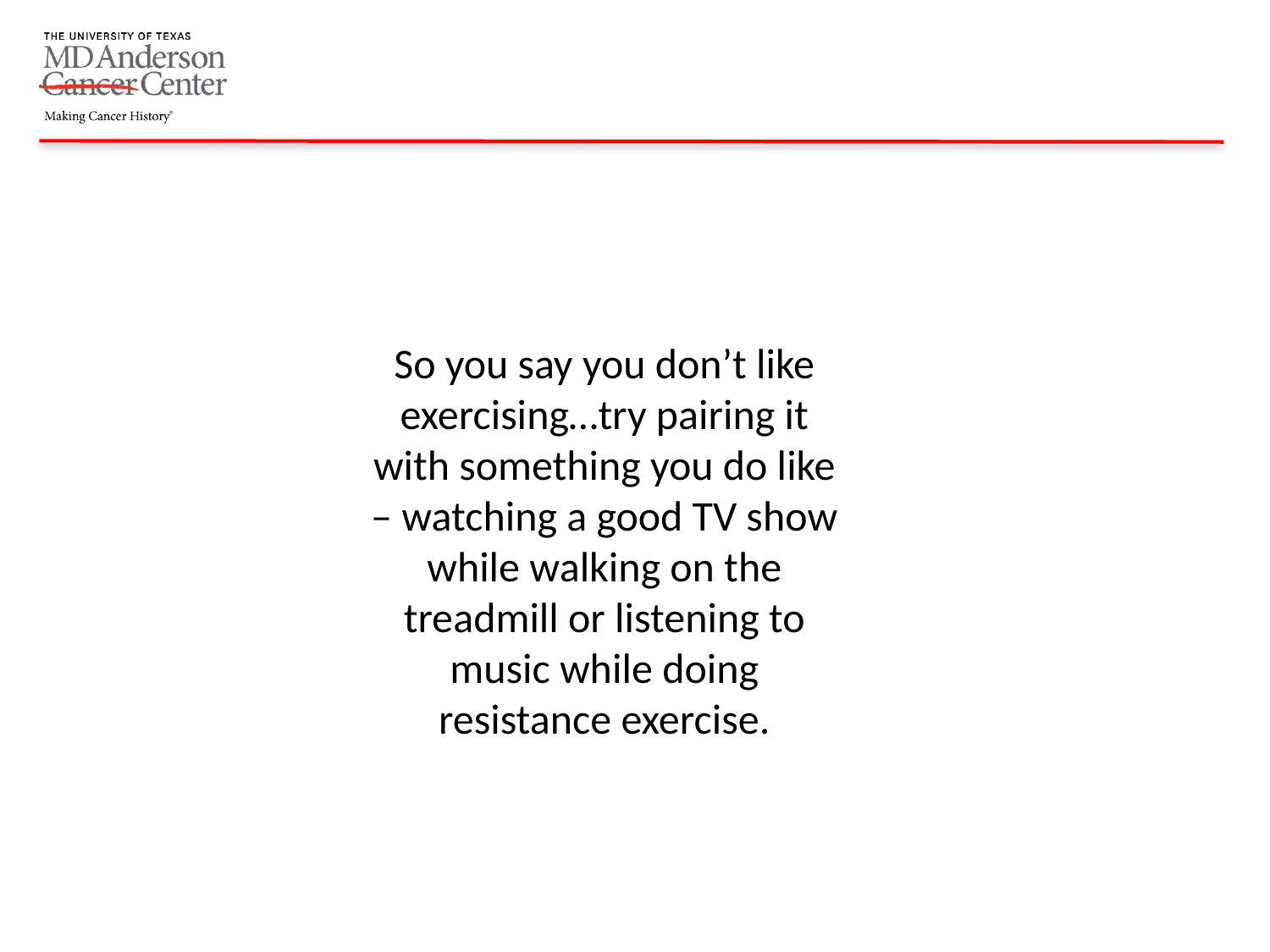

So you say you don’t like exercising…try pairing it with something you do like – watching a good TV show while walking on the treadmill or listening to music while doing resistance exercise.

## Slide 16
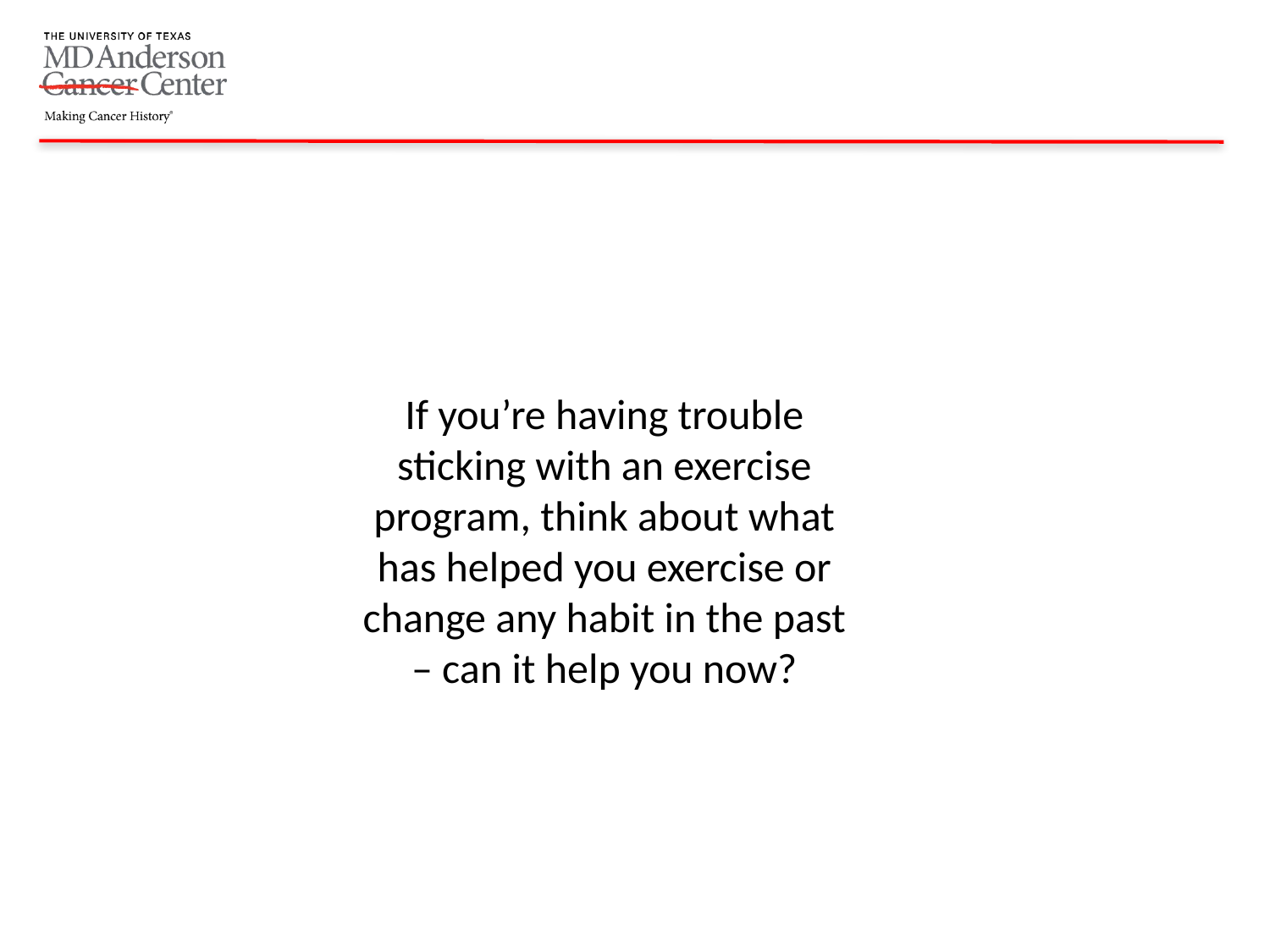

If you’re having trouble sticking with an exercise program, think about what has helped you exercise or change any habit in the past – can it help you now?

## Slide 17
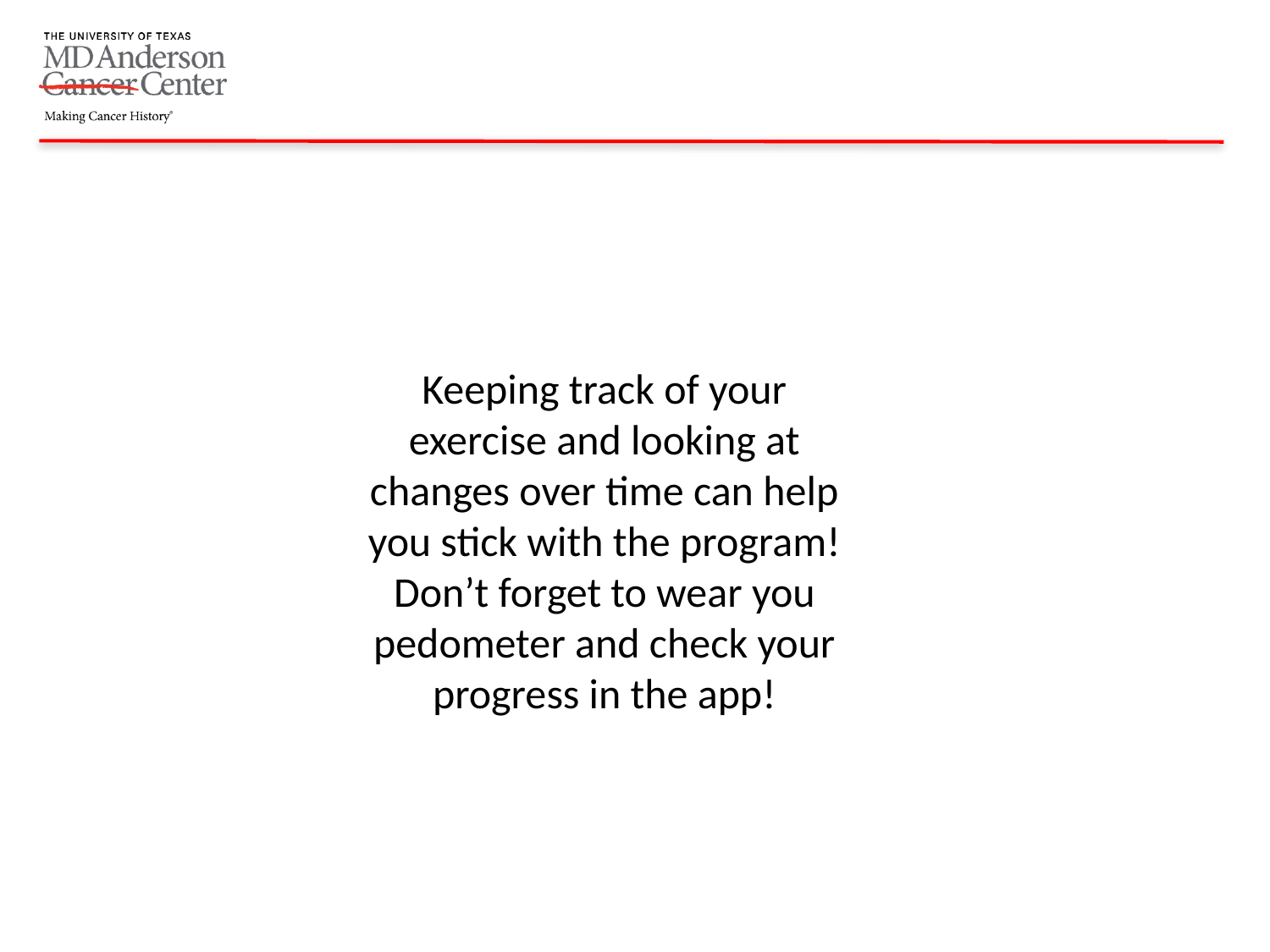

Keeping track of your exercise and looking at changes over time can help you stick with the program! Don’t forget to wear you pedometer and check your progress in the app!

## Slide 18
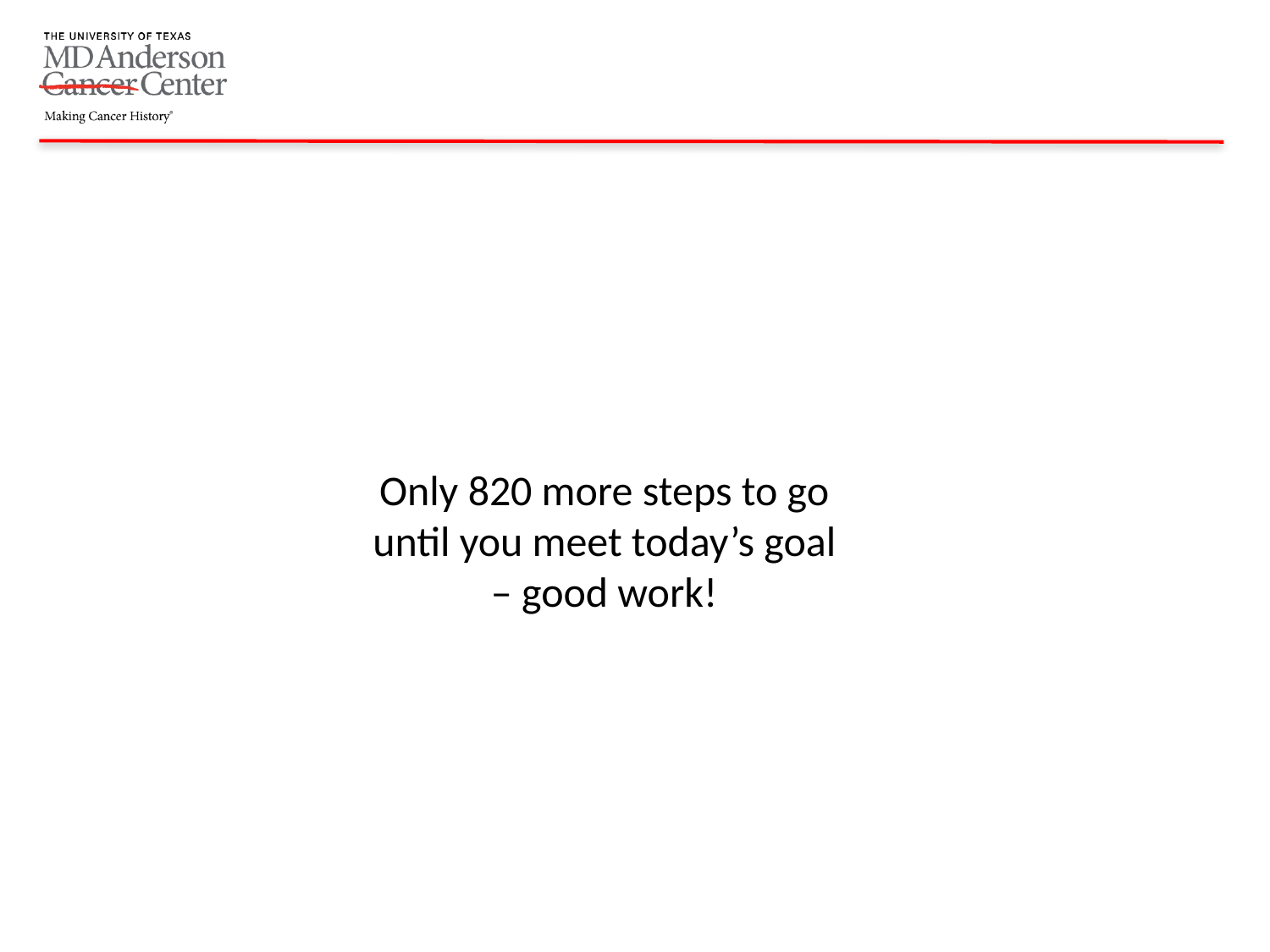

Only 820 more steps to go until you meet today’s goal – good work!

## Slide 19
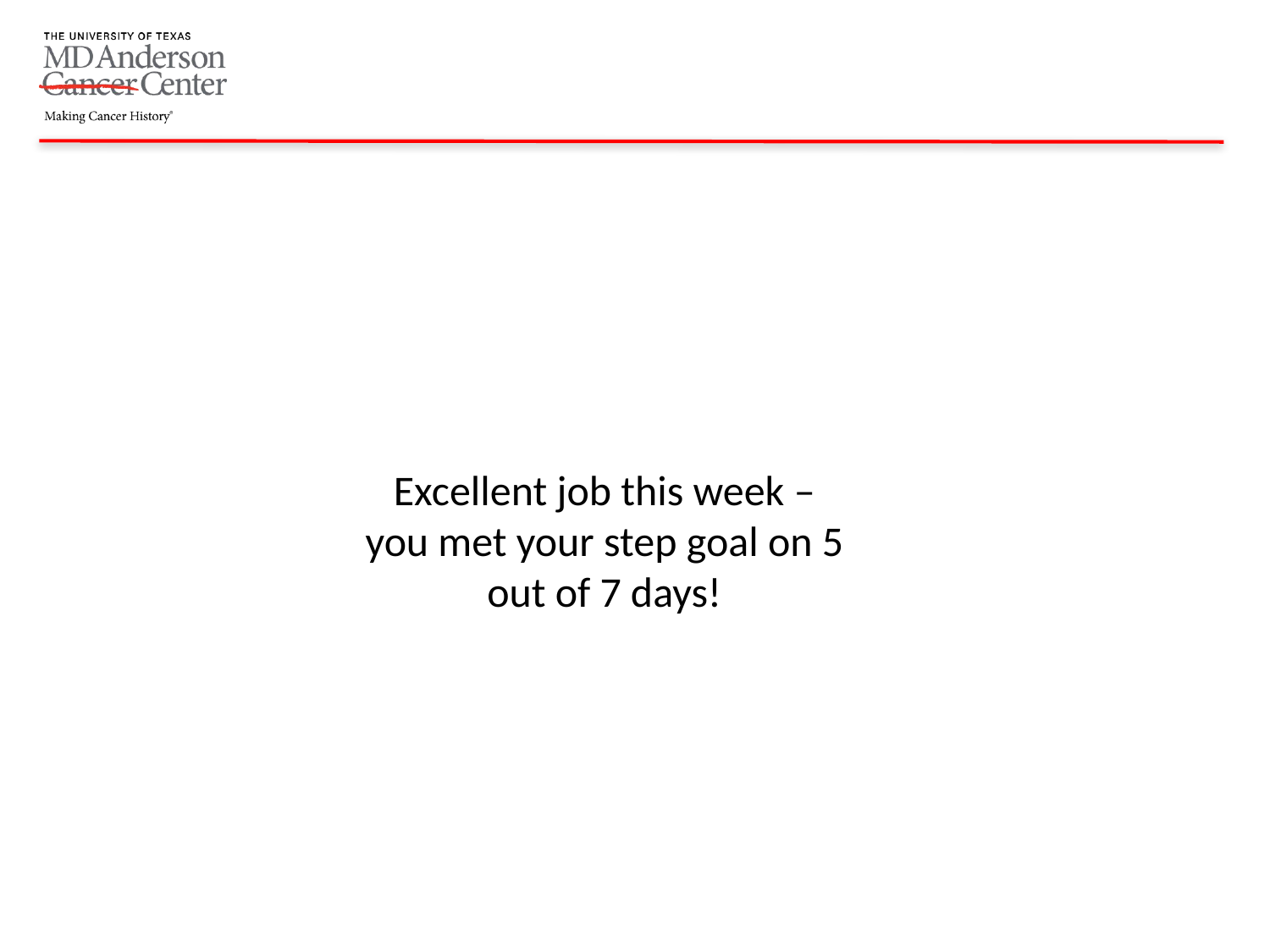

Excellent job this week – you met your step goal on 5 out of 7 days!
